# The Structural Characterisation and DFT-Aided Interpretation of Vibrational Spectra for Cyclo(l-Cys-d-Cys) Cyclic Dipeptide in a Solid State

**DOI:** 10.3390/molecules28155902

**Published:** 2023-08-05

**Authors:** Marcin Witkowski, Damian Trzybiński, Sylwia Pawlędzio, Krzysztof Woźniak, Wojciech Dzwolak, Agata Królikowska

**Affiliations:** 1 Faculty of Chemistry, University of Warsaw, Pasteura 1, 02-093 Warszawa, Poland; 2 Biological and Chemical Research Centre, Chemistry Department, University of Warsaw, Żwirki i Wigury 101, 02-089 Warszawa, Poland; 3 Neutron Scattering Division, Oak Ridge National Laboratory, Oak Ridge, TN 37831, USA

**Keywords:** cyclic dipeptides, X-ray diffraction, structure determination, vibrational spectroscopy, Raman spectroscopy, DFT modeling

## Abstract

Cyclic dipeptides with two intramolecular peptide bonds forming a six-membered 2,5-diketopiperazine ring are gaining significant attention due to their biological and chemical properties. Small changes in the local geometry of such molecules (from *cis* to *trans*) can lead to significant structural differences. This work presents the results of a study of cyclo(l-Cys-d-Cys), a dipeptide comprising two cysteine molecules in opposite chiral configurations, with the functional groups situated at both sides of the diketopiperazine ring. X-ray diffraction (XRD) experiment revealed that the molecule crystallises in the *P*-1 space group, which includes the centre of inversion. The IR and Raman vibrational spectra of the molecule were acquired and interpreted in terms of the potential energy distribution (PED) according to the results of density functional theory (DFT) calculations. The DFT-assisted analysis of energy frameworks for the hydrogen bond network within molecular crystals was performed to support the interpretation of X-ray structural data. The optimisation of the computational model based on three-molecule geometry sections from the crystallographic structure, selected to appropriately reflect the intermolecular interactions responsible for the formation of 1D molecular tapes in cyclo(l-Cys-d-Cys) crystal, allowed for better correspondence between theoretical and experimental vibrational spectra. This work can be considered the first complete structural characterisation of cyclo(l-Cys-d-Cys), complemented via vibrational spectroscopy results with full band assignment aided with the use of the DFT method.

## 1. Introduction

Cyclic dipeptides containing a six-membered 2,5-diketopiperazine ring are an important family of chemical compounds. Naturally occurring diketopiperazines constitute a class of secondary metabolites that are widespread in numerous living organisms [1], including humans [2]. They were marginalised for a long time, considered only as by-products of protein degradation [3,4]. Cyclic dipeptides have been gaining increasing attention due to their constrained geometry, often resulting in improved stability, binding affinity, and specificity compared with their linear counterparts [5]. In addition, their native ability to bind to a wide range of receptors makes them promising templates for drug development [6,7]. Diverse biological properties of 2,5-diketopiperazine-based cyclic dipeptides include antitumor [8], antimicrobial [9], and neuroprotective activity [10], while in chemical applications, they are used as catalysts [11], building blocks for self-assembling hydrogels [12], and polymers with high melting point [13], among others, as reviewed in [14,15,16,17,18].

Cyclic dipeptide isomers can be classified in terms of the configuration of two side chains of the diketopiperazine ring: *cis* (composed of two amino acid units of a single L/D configuration, i.e., L-L or D-D) and *trans* (with alternate L/D chirality). Moreover, the ring can adopt various conformations, which are strongly affected by the properties of amino acids’ side chains. Small substituents tend to form planar structures, such as that of cyclo(Gly-Gly) in a solid state [19], while more bulky residues lead to an out-of-plane conformation, as evidenced by the puckered (twist-boat) structure of the crystalline cyclo(l-Ala-l-Ala) [20] or the less typical flattened chair form, observed, e.g., for cyclo(l-Tyr–l-Pro) [21].

Cyclic dipeptides composed of identical residues are of particular interest, as various conformations and symmetry groups emerge in these molecules. For example, cyclo(d-Ala-l-Ala) exhibits $C_{i}$ symmetry with its nearly planar diketopiperazine ring and the equivalent positions of the two methyl groups [20], and shows a clear contrast resulting from the chirality change to the abovementioned cyclo(l-Ala-l-Ala) isomer with its puckered ring structure. Strong chirality dependence of the structure was also reported for cyclo(Tyr-Tyr) and was explained by stabilising interactions within the O-H∙∙∙O hydrogen bond [22].

Moreover, a proper choice of the structural motif containing a 2,5-diketopiperazine ring unit allows one to control the type of the supramolecular structure emerging from the self-assembly of individual dipeptide monomers. Depending on the type of hydrogen bond involved, crystallographic studies revealed linear, tape structures for planar cyclo(Gly-Gly) and a few other cyclic dipeptides [23] while a layer-type structure was demonstrated for cyclo(l-His-d-His) [24] and cyclo(d-Ala-l-Ala) [25]. Thus, the stereochemistry of dipeptides has a strong influence on the self-assembly process and the resulting architectures: the LL system favours the formation of 1D molecular chains, while LD promotes 2D layers [26]. The use of a combination of hydrogen bonding and aromatic interactions within the molecular crystals of cyclic homodipeptides composed of phenylglycine enabled a rational design of a new material with exceptional mechanical properties [26]. Control over the conformation through the selection of amino acid side chains is also essential for practical applications of dipeptides with the 2,5-diketopiperazine ring, particularly those demanding high selectivity, such as catalytic activity [27], accessibility of metal ion binding sites [28], and smart drug delivery [6,29,30,31].

To the best of our knowledge, this work is the first to present the exact crystal structure of cyclo(l-Cys-d-Cys) in a solid state and a comprehensive analysis of its vibrational spectra, providing complementary information on intermolecular interactions. The selection of naturally widespread cysteine as a building unit of the 2,5-diketopiperazine ring offers exceptional versatility in the design of such materials, due to the presence of a thiol moiety in addition to typical amino acid N- and O-containing units. This opens the route for, e.g., the thiol–ene click reaction, which proceeds with a high yield under mild conditions [32,33,34,35]. Extraordinary chelating properties toward zinc [36], lead [37], silver, nickel, and numerous other ions [38], further enhanced by the rigidity of the six-membered 2,5-diketopiperazine ring, are also anticipated. 

Despite the interest in this family of compounds continuing for more than a century, research on cyclic dipeptides of cysteine is far from exhaustive and still uncovers new issues. A diligent literature review resulted in just a few synthetic protocols for the cyclo(l-Cys-l-Cys) (or cyclo-l-cystine, due to the inevitable creation of intramolecular S-S bond) [39,40,41,42,43,44], as well as several reports of the crystallographic structure [45,46,47] and spectroscopic characterisation, including Raman [48], IR [40,44], nuclear magnetic resonance [49], UV, optical rotatory dispersion, and circular dichroism spectra [50]. Cyclo(l-Cys-l-Cys) was used as an important model for the chiroptical and conformational properties of disulfides [51,52,53]. Gockel et al. concluded that this compound can coordinate zinc(II) ions [54], with binding properties of –SH groups superior to the imidazole ligand in cyclo(l-His-l-His). However, only the binuclear complex (2:2 stoichiometry) was found, suggesting that the localisation of the two thiolate side chains in the ring-shaped molecule was not optimal for the coordination of zinc ions.

Surprisingly, the cyclo(l-Cys-d-Cys) diastereomer seems to have attracted little attention: Our extensive review of the literature yielded only one patent application mentioning this compound [55]. Thus, in this paper, we present a structural description of cyclo(l-Cys-d-Cys) with properties distinctly different than those of the cyclo(l-Cys-l-Cys) due to its *trans* conformation and centrosymmetry. We followed a standard approach for the comprehensive structural characterisation of cyclic dipeptides comprising the analysis of crystallographic X-ray diffraction data and vibrational spectra, together with theoretical simulations, mostly employing density functional theory (DFT) calculations [56,57,58,59,60,61,62]. Additionally, the use of geometry derived from a crystal, utilising one- or three-molecule models in DFT calculations, enhanced the workflow of vibrational analysis and improved the accuracy of the hydrogen bonding model and its effect on vibrational energies.

The complete determination of the crystal structure, as well as the DFT-aided interpretation of the vibrational (IR and Raman) spectra of cyclo(l-Cys-d-Cys) provided a meaningful contribution to the evaluation of the effect of stereochemistry on tailoring the structure of 2,5-diketopiperazine-based dipeptides toward specific applications.

## 2. Results and Discussion

### 2.1. Crystallographic Analysis

**Figure 1:**
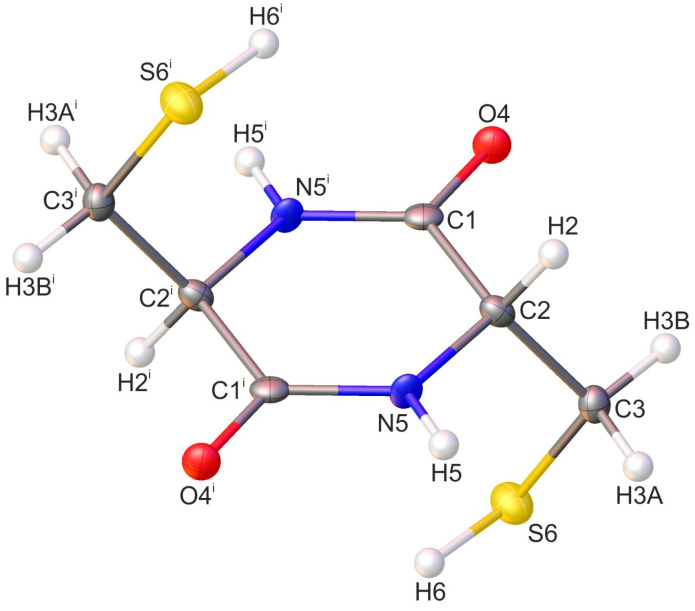
Molecular structure of cyclo(l-Cys-d-Cys) compound with crystallographic atom numbering (the asymmetric unit of the crystal lattice consists of the part of the molecule with the atoms not labelled with a symmetry code). Hydrogen atoms are in white, oxygen atoms in red, sulfur in yellow, and nitrogen in blue. The same atom numbering was used to present the results of the PED analysis. Displacement ellipsoids are drawn at the 50% probability level. The H-atoms are shown as small spheres of an arbitrary radius. Symmetry code: (i) −x + 1, −y, −z + 1.

The identity of the investigated compound was confirmed via single-crystal X-ray diffraction (XRD) analysis. Cyclo(l-Cys-d-Cys) crystallises in a triclinic *P*-1 space group with half of the molecule in the asymmetric unit of the crystal lattice (Figure 1). The crystal and structural refinement details are presented in Table S1 (Supplementary Material). The list of bond lengths, valence, and torsion angle values is provided in Tables S2, S3, and S4 of Supplementary Materials, respectively.

The diketopiperazine (DKP) ring is planar, with the atoms of the ring having a standard deviation of 0.003 Å from the mean plane. This planar conformation is similar to that observed in unsubstituted 2,5-diketopiperazine [19,63,64,65] or cyclo(l-Ser-l-Ser) [66]. A more in-depth comparison of the conformation to other cyclic dipeptides is presented in Table 1, which shows that the virtually ideal planarity of the DKP ring is uncommon across cyclic dipeptides, including those constituting amino acids of opposing chirality. The thiol side chains are twisted above and below the ring in such a way that the overall conformation of the cyclo(l-Cys-d-Cys) molecule is S-shaped. The values of the peptide torsional angles *ω*, *φ,* and *ψ* are 0.9(5)°, 0.8(5)°, and 0.8(4)°, respectively.

**Figure 2:**
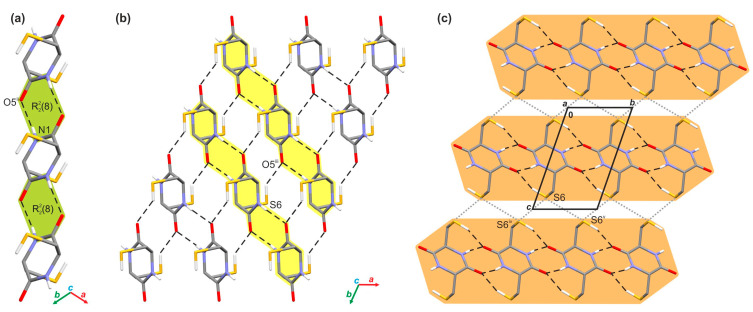
Supramolecular architecture of the crystal of investigated compound: (a) H-bonded tape of molecules running along *[101]* direction (the R^2^_2_(8) motifs are highlighted in green); (b) section of infinite layer built of H-bonded tapes of molecules spreading along *[101]* plane (single tapes are highlighted in yellow); (c) general view of the layered arrangement of molecules (single layers are highlighted in orange), viewed along the crystallographic *a*-direction. The hydrogen bonds are represented by the black dashed lines and the S···S contacts by the grey dotted lines. The H-atoms not participating in the above intermolecular interactions were omitted for clarity. Symmetry codes: (ii) *x* − 1, *y* − 1, *z*; (iii) *x*, −*y* + 1, *z*; (iv) −*x* + 2, −*y*, −*z* + 2; (v) −*x* + 2, −*y* + 1, −*z* + 2.

The crystallographic analysis allowed for an understanding of the supramolecular architecture of investigated crystal. In this case, adjacent molecules of cyclo(l-Cys-d-Cys) are held together by a network of the N-H···O (*d*(D···A) = 2.843(3) Å, <(D-H···A) = 168(3)°) hydrogen bonds in one-dimensional tapes running along the *[101]* direction (Figure 2a). The hydrogen bonds within these supramolecular assemblies form the R^2^_2_(8) ring motif (Figure 2a). The presence of similar tapes was reported in the past for other symmetrically substituted diketopiperazines [91]. In the next step, adjacent molecules in neighbouring tapes further interact with themselves via the S-H···O hydrogen bonds (*d*(D···A) = 3.387(2) Å, <(D-H···A) = 144(2)°) (Table S5 in Supplementary Materials), which results in the appearance of an infinite layer of molecules spreading along the *[101]* plane (Figure 2b). Such formed layers also interact with each other through the network of bifurcated S···S contacts (*d*(S···S) = 3.590(2) and 3.556(2) Å) that involve neighbouring molecules from adjoining layers (Figure 2c).

### 2.2. DFT Studies of the Cyclo(l-Cys-d-Cys) Molecule

#### 2.2.1. Investigation of the Interaction Energy in the Crystal

**Figure 3:**
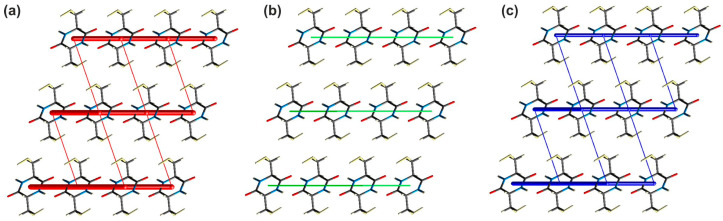
Energy frameworks of cyclo(l-Cys-d-Cys) representing the (**a**) electrostatic, (**b**) dispersive, and (**c**) total energy viewed along the crystallographic *a* direction. The tube size is 30 arbitrary units. The radius of the cylinder is proportional to the magnitude of the pairwise interaction energy. Interactions of energy values less than 10 kJ/mol were omitted for clarity.

The analysis of the calculated energy values for respective pairwise interactions (Table S6) shows that the most energetically relevant interactions in the analysed crystal network are the hydrogen bonds. Thus, the total interaction energy values within the pair of molecules engaged in the N−H···O and S−H···O contacts are −71.4 and −40.3 kJ/mol, respectively. In the case of the S−H···O contacts, the value is smaller; however, it cannot be overlooked that the dimer modeling the N−H···O bonding is tied together by two hydrogen bonds. Some differences can be noted, considering the nature of the above contacts. Theoretical calculations reveal that the N−H···O interactions are mostly Coulombic, while in the case of the S−H···O hydrogen bonds, the Coulombic and dispersive contributions are almost the same. Moreover, it can be seen that the intermolecular S···S contacts assisting the hydrogen bonds in the cyclo(l-Cys-d-Cys) crystal are significantly weaker (−6.3 and −3.0 kJ/mol) than the hydrogen bonding interactions. In the case of stronger contact (S6···S6^iv^; (iv) −*x* + 2, −*y*, −*z* + 2), the Coulombic contribution dominates the dispersive one, while for the weaker one (S6···S6^v^; (v) −*x* + 2, −*y* + 1, −*z* + 2), the opposite tendency is observed. In addition to the above interactions, the crystal lattice is stabilised via a network of dispersive contacts with total energy values of −19.0, −14.7, and −6.3 kJ/mol, respectively. The strength and nature of individual pairwise interactions within the investigated crystal are additionally shown in the form of energy frameworks (Figure 3).

#### 2.2.2. Optimising the Model of a Single Cyclo(l-Cys-d-Cys) Molecule

The XRD-derived experimental structure of cyclo(l-Cys-d-Cys) was used as a starting point for the optimisation of the computational model of the cyclo(l-Cys-d-Cys) single molecule (see Figure S1 for comparison of optimized theoretically and XRD-originating structure) and, subsequently, its calculated vibrational spectra. However, obtaining a valid computational model that corresponded to a minimum on the energy hypersurface was not straightforward. These problems are identified and described in Section S1.2 of Supplementary Materials. The final valid model that is used and discussed in the results presented here has all harmonic frequencies calculated to be real. This model was obtained by first optimising a system composed of three molecules involved in N-H···O hydrogen bonding at the B3LYP/May-cc-pVDZ level of theory (within the $C_{i}$ symmetry) and then reoptimising the middle molecule using any of the augmented cc-pVDZ basis sets. The stability of the wave function upon the release of the symmetry constraints was verified. Unless stated otherwise, the results discussed here were obtained using this model at the B3LYP/Aug-cc-pVDZ level of theory.

The three-molecule model in which cyclo(l-Cys-d-Cys) molecules interact via two pairs of the N-H···O hydrogen bonds was flawlessly optimised at the B3LYP/Aug-cc-pVDZ level of theory in $C_{i}$ symmetry using the positions of atoms derived from the XRD structure (Figure S2a). The model incorporating the S-H···O hydrogen bonds was obtained in the same manner (Figure S2b), but required the addition of Grimme’s D3 empirical dispersion [92] in order to converge into the designated geometry.

#### 2.2.3. Structure of the Optimised Model

The geometry of the optimised structure of a single cyclo(l-Cys-d-Cys) molecule at the B3LYP/Aug-cc-pVDZ level, superimposed on the geometry obtained from the XRD data, is presented in Figure S1, while the calculated basic parameters of the structure are compared with the experimental values in Tables S2–S4. In general, the optimisation process did not alter the geometry significantly, and the model can be considered a reasonable approximation of the cyclo(l-Cys-d-Cys) structure present in the solid state. 

Similar conclusions can be drawn for the three-molecule models, visualised in Figure S2. Their structures are closely related to the experimental XRD-derived positions; thus, the intermolecular interactions within them can be expected to follow the experimental system.

The atom positions of all the models are disclosed in Section S2.1. of Supplementary Materials.

#### 2.2.4. Conformational Analysis

To put the molecular geometry from the XRD into a broader context, a complete conformational analysis of the system was also performed. The systematic rotor search implemented in Avogadro yielded nine conformers of cyclo(l-Cys-d-Cys) (denoted Conf1 to Conf9). The atomic coordinates of all conformers are reported in Section S2.2. of Supplementary Materials. All the predicted conformers were reoptimised at the B3LYP/Aug-cc-pVDZ level of theory without empirical dispersion, resulting in eight distinct structures (two different initial conformers converged to one final state), all of which were verified to be at the minimal point of the energy hypersurface using vibrational analysis. The coordinates of the atoms in these models are presented in Section S2.3 of Supplementary Materials.

It was revealed that the conformer evidenced in the experimental solid-state structure of cyclo(l-Cys-d-Cys) was not found through the systematic search performed using Avogadro. Furthermore, this conformation, with the computational methods and procedures used (the single molecule in the gas phase surrounded by vacuum), had significantly higher energy than the supposedly best structure among the optimised conformers. The difference of about 4.00 kJ/mol can be attributed to the intermolecular hydrogen bonding stabilising the structure, which is absent in this model. A detailed comparison of the energy values obtained for the various conformers is presented in Table S7 in Supplementary Materials.

### 2.3. Vibrational Spectroscopy

**Figure 4:**
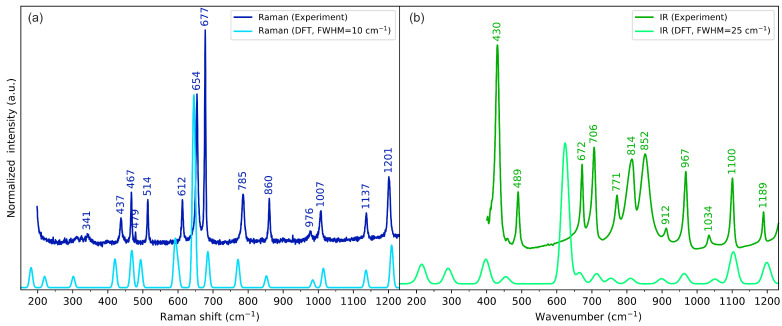
Experimental (dark-coloured lines) and theoretical (light-coloured lines) (**a**) Raman and (**b**) IR spectra of cyclo(l-Cys-d-Cys) in the low-wavenumber range. Theoretical spectra were calculated using a one-molecule in-vacuum model at the B3LYP/Aug-cc-pVDZ level of theory.

**Figure 5:**
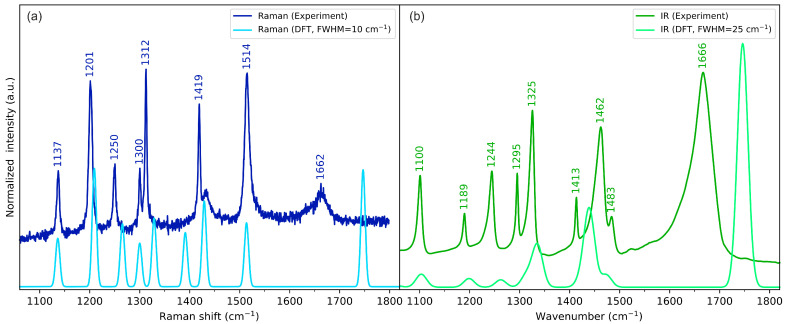
Experimental (dark-coloured lines) and theoretical (light-coloured lines) (**a**) Raman and (**b**) IR spectra of cyclo(l-Cys-d-Cys) in the medium-wavenumber range. Theoretical spectra were calculated using a one-molecule in-vacuum model at the B3LYP/Aug-cc-pVDZ level of theory.

**Figure 6:**
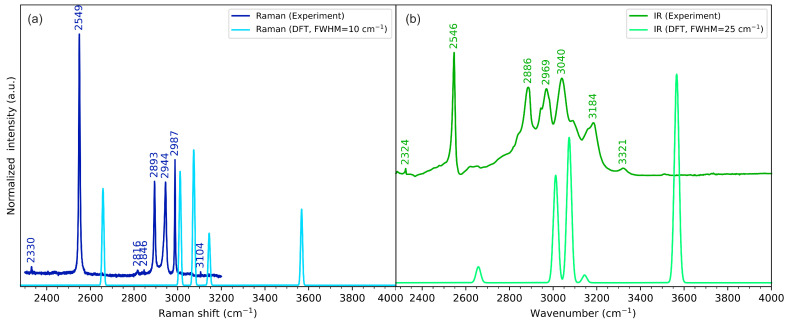
Experimental (dark-coloured lines) and theoretical (light-coloured lines) (**a**) Raman and (**b**) IR spectra of cyclo(l-Cys-d-Cys) in the high-wavenumber range. Theoretical spectra were calculated using a one-molecule in-vacuum model at the B3LYP/Aug-cc-pVDZ level of theory.

The experimental and calculated vibrational (IR and Raman) spectra of cyclo(l-Cys-d-Cys) in the low-, medium-, and high-wavenumber ranges are compared in Figure 4, Figure 5, and Figure 6, respectively. The plots showing the full-range overview of the spectra are shown in Figures S3 and S4 for IR and Raman, respectively. All the results in the figures are presented for the computational gas-phase model of a single cyclo(l-Cys-d-Cys) molecule calculated at the B3LYP/Aug-cc-pVDZ level of theory. The full vibrational assignment based on the PED analysis is shown in Table S8, while the summary of the most prominent experimental bands is presented in Table 2.

In general, the single-molecule model is in good agreement with the experimental spectra. The largest discrepancies were observed for bands originating from vibrations of atoms involved in intermolecular interactions, which were not included in the computational model. Such differences are discussed in the following paragraphs in the context of DFT calculations for models comprising three cyclo(l-Cys-d-Cys) molecules. 

In our system, the three-molecule model is the simplest one that allows for the computational vibrational analysis of a molecule surrounded by other interacting molecules from both sides, as deduced from XRD analysis. In the assumed model, the vibrations of the interacting groups (e.g., amino group and carbonyl group) can be divided into groups localised on the outer molecules (e.g., for atoms not involved in the hydrogen bonding and thus virtually absent in the solid state) and groups localised on the central molecule and the adjacent groups directly interacting with it. Ultimately, only the vibrations of groups involved in intermolecular interactions are relevant for the assignment of the experimental bands, and only these modes will be generally discussed in more detail.

#### 2.3.1. N-H Stretching Vibrations

According to DFT calculations, in the isolated cyclo(l-Cys-d-Cys) molecule, there are two bands that can be attributed to the ν_N-H_ vibrations: symmetric stretching at 3568 cm^−1^ (active in Raman) and antisymmetric stretching at 3566 cm^−1^ (active in IR). These vibrations can be assigned to the experimental bands at 3160 cm^−1^ and 3185 cm^−1^ in Raman and IR, respectively, in line with the literature assignment for cyclo(l-Met-l-Met) [80]. The energy of these vibrations is obviously vastly overestimated in the computational model, as the single-molecule model does not include hydrogen bonding. The three-molecule model predicts the in-phase stretching vibrations of the N-H groups involved in the hydrogen bonding at 3192 cm^−1^ (symmetric, Raman-active vibration) and 3194 cm^−1^ (antisymmetric, IR-active vibration). These DFT-calculated vibrational energies are much closer to the experimental values, proving that hydrogen bonding substantially influences the N-H vibrations. The IR spectrum also includes a very weak signal at 3321 cm^−1^. Weak bands appearing at similar energy are sometimes assigned to the N-H stretching vibration (e.g., the 3328 cm^−1^ band of cyclo(Gly-l-Val) [59], the 3327 cm^−1^ band of cyclo(l-His-l-Phe) [60], 3329 cm^−1^ band in cyclo(Gly-Leu) [61], or the 3320 cm^−1^ band of cyclo(l-Ala-l-His) [62]), while other times they are not assigned at all (e.g., the 3328 cm^−1^ band in cyclo(Gly-Gly), 3322 cm^−1^ in cyclo(l-Ala-l-Ala), or 3326 cm^−1^ in cyclo(l-Ala-Gly) [57]). As the cyclo(l-Cys-d-Cys) system is centrosymmetric and thus restricts the normal modes to be active only in either Raman or IR spectra, the number of available normal modes that can be assigned to the experimental bands is even smaller. Thus, for consistency with the symmetry rules, in our assignment, this band is not explicitly assigned to the ν_N-H_ normal mode. Instead, we tentatively attribute it to the overtone of the very strong 1667 cm^−1^ band (see Figure 5). This assignment could be improved by performing the isotopic substitution, which is beyond the scope of this work. At the same time, the reports on the results of such experiments in other cyclic dipeptides are not unequivocal, as the band seems to remain present for cyclo(l-Ala-l-Ala) after an isotopic exchange but disappears for some other dipeptides [57].

Contrary to the DFT predictions, the observed Raman band due to ν_N-H_ is very weak (Figure 5); however, this can be again rationalised by considering that the hydrogen atom of the amino group is involved in hydrogen bonding. As the vibrational spectra were collected for the samples in a solid state, the network of molecules can have various local defects that lead to changes in the local intermolecular interactions, affecting the energy of this vibration and, as a result, broadening the peak. The respective IR signal is also broader than others, but its intensity is sufficient to easily resolve it.

#### 2.3.2. C=O Stretching Vibrations

Generally speaking, the carbonyl group stretching vibrations can be analysed in analogy to the N-H group stretching vibrations. The one-molecule model estimated the energy of these vibrations at 1746 cm^−1^ and 1747 cm^−1^ (antisymmetric and symmetric vibrations, respectively), but the plausible experimental bands corresponding to these vibrations appear as relatively broad features at 1667 cm^−1^ and 1664 cm^−1^ in IR and Raman, respectively (Figure 5). As the crystallographic analysis shows that the DKP ring is planar, these values can expand the systematic analysis of the dependence between the conformation of the ring and the energy of the vibrations provided by Palacin et al. [91]. The IR and Raman spectra of cyclic dipeptides of two different amino acids generally exhibit a doublet resulting from the stretching of two non-equivalent carbonyl groups in the diketopiperazine ring [59,60,62]. The splitting into doublet also appears for some cyclic dipeptides composed of two molecules of the same amino acid with the same chirality (including, e.g., cyclo(l-Asp-l-Asp) [58] and cyclo(Gly-Gly) [93]) due to the presence of more than one molecule in the unit cell, contrary to cyclo(l-Ala-l-Ala) that includes only one molecule in its unit cell [57]. Finally, a previous report on cyclo(d-Ala-l-Ala) shows the group splitting as well, again rationalising it with the occurrence of two molecules in the unit cell [94]. In the case of the compound studied here, the unit cell only contains half of the molecule; thus, no group splitting is observed.

This substantial shift between the energy value of the ν_C=O_ vibration calculated for the one-molecule model and the experimental spectra can be a result of the N−H···O hydrogen bonding since the three-molecule model that includes these interactions predicts the bands originating from in-phase carbonyl stretching vibrations at 1702 cm^−1^ and 1705 cm^−1^ (antisymmetric and symmetric vibrations, respectively). The PED analysis of the three-molecule model reveals that this mode is highly coupled with the δ_HNC_ vibrations, which in turn increase their energy compared with the one-molecule model. This type of coupling was postulated for a variety of cyclic dipeptides and proved using deuteration studies [56]. The more in-depth analysis of these modes is challenging, however, as the decomposition reported using the VEDA4 (v. 4e1) software is limited to vibrations with no less than 10% of energy contribution to a particular mode. As the number of vibrations involved in modes rapidly increases with the number of atoms in the model, only a high-level overview can be reliably provided. Nonetheless, both the energy shift of the DFT-predicted modes and the involvement of the δ_HNC_ vibration in the mode itself prove the significance of considering intermolecular interactions for the energy values and interpretation of carbonyl group stretching vibrations.

However, the N−H···O hydrogen bonding can describe only part of the discrepancy between the single-molecule DFT model and the experimental results. The crystal structure and the calculations presented in Section 2.2.1. reveal that the S−H···O hydrogen bonding can also be considered an important intermolecular interaction influencing the energy of the carbonyl group stretching vibration. The three-molecule model (visualised in Figure S2b) developed to address these interactions predicts the ν_C=O_ vibrations at the energy of 1720 and 1728 cm^−1^ (symmetric and antisymmetric vibrations, respectively). Thus, the cumulative effect of all the intermolecular interactions is a decrease in the experimental energy of the stretching vibration.

Furthermore, the Raman band due to carbonyl stretching vibration is again weak and broad, suggesting that there is some variation in intermolecular interactions in the sample, typical of a non-monocrystalline state.

#### 2.3.3. S-H Stretching Vibrations

The S-H stretching vibrations were assigned to the bands at 2547 cm^−1^ and 2549 cm^−1^ in IR and Raman, respectively. The one-molecule model again vastly overestimated the energy of these bands, calculating both of them at 2658 cm^−1^. Again, the discrepancy results from neglecting the intermolecular interactions, as the three-molecule model that includes the S−H···O hydrogen bonding estimated the energy of these vibrations at 2617 cm^−1^, much closer to the experimental values.

#### 2.3.4. Skeletal Vibrations

The skeletal vibrations of cyclo(l-Cys-d-Cys) are generally reproduced fairly well using the one-molecule model, particularly for atoms that are not considerably involved in intermolecular interactions. Approximate vibrational assignments will be given below, but since these modes are generally delocalised across the molecule, only the results of the PED analysis in Table S8 are able to provide a comprehensive description of the system. Nonetheless, this interpretation can serve as a high-level approximation and a starting point for a more in-depth analysis. In descending energy order, there is a ring deformation mode mainly composed of the in-plane motion of nitrogen atoms: combined N-C stretching and HCN bending. The in-phase and out-of-phase modes relative to the inversion centre were assigned to a weak Raman band at 1300 cm^−1^ and a strong IR band at 1325 cm^−1^, respectively (and their DFT-calculated equivalents at 1300 cm^−1^ and 1335 cm^−1^, respectively). Subsequently, there are hydrogen wagging vibrations (localised on all the hydrogen atoms except for the thiol groups): one with methylene wagging and methine group hydrogens moving in-phase, which were assigned to the Raman band at 1312 cm^−1^ and the IR band 1295 cm^−1^ (calc. 1329 cm^−1^ and 1313 cm^−1^, respectively), and one with hydrogens of C-H moving in counterphase, which were assigned to the Raman band at 1250 and the IR band at 1244 cm^−1^ (calc. 1265 cm^−1^ and 1262 cm^−1^, respectively). Next, the carbon–hydrogen twisting vibrations in the methylene group coupled with the in-plane bending of the HCN fragment were attributed to the Raman band at 1201 cm^−1^ and IR signal at 1190 cm^−1^ (calc. 1209 cm^−1^ and 1199 cm^−1^, respectively). Subsequently, we assigned the N-C stretching vibrations to the Raman peak at 1137 cm^−1^ and IR peak at 1100 cm^−1^ (calc. 1136 cm^−1^ and 1103 cm^−1^, respectively). The IR signal at 1034 cm^−1^ and Raman signal at 1007 cm^−1^ were attributed to C_ring_-C_methylene_ stretching vibrations (calc. 1051 cm^−1^ and 1014 cm^−1^, respectively). Following that, the methylene group rocking coupled with CSH bending manifested itself with bands at 976 cm^−1^ in Raman and 967 cm^−1^ in IR (calc. 984 cm^−1^ and 963 cm^−1^, respectively) for in-phase vibrations and at 814 cm^−1^ in IR and 785 cm^−1^ in Raman (calc. 810 cm^−1^ and 771 cm^−1^, respectively) for vibrations in counterphase. 

Out-of-plane ring deformations were attributed to Raman bands at 862 cm^−1^ and 679 cm^−1^ (calc. 852 cm^-1^ and 685 cm^−1^, respectively), as well as IR signals at 912 cm^−1^, 772 cm^−1^, and 707 cm^−1^ (calc. 899 cm^-1^, 754 cm^−1^, and 713 cm^−1^, respectively). Notably, in this model, the experimental Raman band at 679 cm^−1^ (calc. 685 cm^−1^) exhibited a significantly higher intensity than that predicted with DFT (both with one- and three-molecule models). Moreover, this intensity was not consistent across the sample, showing different relative intensity ratios with respect to the 656 cm^−1^ signal (lower or higher than 1), depending on the location. Finally, the IR band at 672 cm^−1^ and the Raman band at 654 cm^−1^ were assigned to the strongly delocalised S-C and C-C stretching vibrations, coupled with out-of-plane N-H bending (calc. 665 cm^−1^ and 645 cm^−1^, respectively), while the 431 cm^−1^ IR band was assigned to OCN bending (calc. 398 cm^−1^).

## 3. Experimental Procedure

### 3.1. Materials and Methods

Cyclo(l-Cys-d-Cys) (preferred IUPAC name: (3*R*,6*S*)-3,6-bis(mercaptomethyl)piperazine-2,5-dione, purity ≥ 95%) was custom-synthesised by Bachem (Bubendorf, Switzerland) and used as received without any purification apart from the crystallisation studies. Acetonitrile (p.a.) was purchased from POCh (Gliwice, Poland). Ultrapure water obtained from a Milli-Q system (Millipore, Burlington, MA, USA) was used throughout all the experiments, and its specific resistivity was controlled at 18.2 MΩ·cm.

### 3.2. Raman Spectroscopy

The Raman spectra of solid cyclo(l-Cys-d-Cys) were acquired using a LabRAM HR800 (Horiba Jobin Yvon S.A.S., Palaiseau, France) coupled to an Olympus BX41 (Olympus, Tokyo, Japan) confocal microscope. Vibrations were excited at room temperature using a diode-pumped frequency-doubled Nd:YAG laser providing 532 nm radiation (100 mW power at the head, less than 5 mW at the sample). The instrument was configured in the back-scattering mode, and the signal was analysed and collected with 1800 grooves/mm diffraction grating (unless stated otherwise) and a Peltier-cooled (−70 °C) CCD detector (1024 × 256 pixels). The excitation beam was focused on the sample surface using 50× long working distance Olympus objective lenses. The spectrometer was calibrated using the 520 cm^−1^ Raman band of crystalline silicon. The experimental Raman spectra were baseline-corrected using the LabSpec 5.45.09 software, applying the implemented automatic procedure of the polynomial fitting of the sixth degree.

### 3.3. IR Spectroscopy

A solid-state sample of cyclo(l-Cys-d-Cys) was studied via Fourier-transform IR spectroscopy using a Nicolet iS50 (Thermo Scientific, Waltham, MA, USA) spectrometer. The spectra were acquired using attenuated total reflectance (single reflection diamond ATR accessory) mode in the 400–4000 cm^−1^ region. The nominal spectral resolution of the measurement was 2 cm^−1^.

### 3.4. Single-Crystal Growth

Single crystals of cyclo(l-Cys-d-Cys) were obtained via slow solvent evaporation from the solution under ambient conditions. About 1 mg of cyclo(l-Cys-d-Cys) was dissolved in 4 mL of an acetonitrile–water 1:1 (*v*/*v*) mixture. The solution was left in the fume hood until the solvent evaporated completely. The generated crystals were initially analysed using an Olympus BX41 optical microscope to select a suitable specimen for further crystallographic studies.

### 3.5. X-ray Diffraction Data Collection and Structural Refinement

A good-quality single crystal of the investigated compound was selected for the X-ray diffraction experiment at T = 100(2) K. The crystal was mounted with paratone-N oil using the MiTeGen micromount. Diffraction data were collected on an Agilent Technologies (Santa Clara, CA, USA) SuperNova Dual Source diffractometer with MoKα radiation (*λ* = 0.71073 Å) using the CrysAlis PRO software (version 39.46) [95]. The analytical numeric absorption correction using a multifaceted crystal model based on the expressions derived by Clark and Reid was applied [96]. The structural determination procedure was carried out using the SHELX package. The structure was solved with the intrinsic phasing method using the SHELXT program [97], and then successive least-square refinement was carried out based on the full-matrix least-square method on *F*^2^ using the SHELXL program [98]. The H atoms linked to the N and S atoms were located on the Fourier difference electron density map and refined to *U*_iso_(H) = 1.2*U*_eq_(N). The remaining H atoms were positioned geometrically with C–H equal to 0.97 and 0.98 Å for methine and methylene, respectively, and constrained to ride on their parent atoms with *U*_iso_(H) = 1.2*U*_eq_(C). The molecular interactions in the crystal of the investigated compound were identified using the PLATON program [99]. Figure for this publication were prepared using Olex2 and Mercury programs [100,101]. 

### 3.6. Theoretical Calculations of Pairwise Intermolecular Interaction Energies

The selected energies of pairwise intermolecular interactions in the crystal of investigated compound were calculated using CrystalExplorer 21.5 [102,103]. The wave-function calculations were performed using Becke’s three-parameter functional [104,105] incorporating the non-local correlation as defined by the Lee–Yang–Parr expression [106] (B3LYP) and the 6-31G(d,p) basis set [107,108,109,110,111] along with Grimme’s D2 dispersion corrections [112] using Gaussian16 software (revision C.01) [113]. Single-point calculations were performed for each example of a pair of interacting molecules in the crystal. The obtained CE-B3LYP model energy enabled us to obtain accurate values of electrostatic, polarisation, and repulsion energies and represent energies between interacting molecules as energy frameworks [114].

### 3.7. DFT Calculations

All calculations were performed using the Gaussian 16 C.01 software suite [113]. Density functional theory was used as a primary model [115,116,117]. B3LYP was used as a functional of choice, as it has been successfully utilised for similar applications in the literature [118,119,120]. Calculations were typically performed with a Dunning-type double-zeta basis set family (cc-pVDZ) [121,122]. The augmentation of the fundamental basis set with diffuse function either on all atoms (Aug-cc-pVDZ) [123] or as described by Truhlar et al. in their calendar basis sets (May-, Jun-, and Jul-cc-pVDZ) [124] was used whenever necessary.

The initial geometry for optimisation processes was developed in one of the following two ways: The first approach involved using conformer generation based on a systematic rotor search as implemented in the Avogadro software (version 1.2.0) [125,126]. In the second approach, the initial geometry was derived from the XRD data. One or three molecules were extracted from the crystallographic structure. The molecules were selected so that they were involved in intermolecular hydrogen bonding, with two pairs of either S−H···O or N−H···O hydrogen bonds. The one-molecule model and the three-molecule model that included the N−H···O hydrogen bonds were optimised without taking into account empirical dispersion, while the three-molecule model that included the S−H···O hydrogen bonds was optimised using Grimme’s D3 empirical dispersion correction [92].

Optimisation was performed for each initial geometry. The energy of harmonic oscillations was calculated for each final geometry [127,128,129], together with the respective theoretical IR intensities and Raman activities. The obtained vibrational energies were not scaled, although in general, it can improve the agreement between calculated and experimental bands [130]. The transformation from Raman activities to excitation-dependent Raman intensities was conducted according to the method described in our earlier studies [131,132]. The relevant calculations were performed using house-made scripts based on the SciPy library [133]. The calculated Raman and IR spectra were prepared using Gaussian-type signals, with FWHM of 10 and 25 cm^−1^, respectively. For visualisation purposes, the computed spectra were normalised via multiplication with a constant chosen in such a way that the global intensity of the theoretical spectra was similar to the intensity of the experimental spectra.

The assignment of vibrational modes for the selected systems was carried out by means of potential energy distribution (PED) analysis using VEDA4 software (version 4e1) [134,135]. The visualisations of the optimised structures superimposed onto the positions derived from the XRD experiments were prepared using Facio software (version 23.1.5.64) [136].

## 4. Conclusions

This work describes the crystal structure and vibrational (IR and Raman) spectra in the solid state of a cyclic dipeptide composed of two cysteine molecules of opposing chirality, cyclo(l-Cys-d-Cys). Cyclo(l-Cys-d-Cys) crystallises in the *P*-1 space group, with half of the molecule in the unit cell. The diketopiperazine ring in the molecule is almost exactly planar, whereas the whole molecule assumes an S-shape in the crystal. Numerous intermolecular interactions can be found within the crystal, among which the N−H···O and S−H···O hydrogen bonds were calculated to be the strongest. The molecules involved in N−H···O hydrogen bonding are arranged into one-dimensional molecular tapes that run along the *[101]* direction. This type of architecture in a solid is not intuitive, as usually, cyclic LD-type dipeptides favour the formation of 2D layers. Such one-dimensional hydrogen-bonded tapes in the crystalline solid are intriguing materials as a scaffold to further design the structure and molecular properties of solids.

Curiously, the conformation assumed with the cyclo(l-Cys-d-Cys) molecule in the crystal was not found using a standard systematic rotor search procedure for conformer identification.

The atomic positions derived from the XRD experiment were used as an initial geometry for optimisation in the DFT model of cyclo(l-Cys-d-Cys). A model comprising one molecule was used for the assignment of the IR and Raman spectra acquired for the sample in the solid state. A high degree of agreement between the DFT-calculated and the experimental spectra allowed for a comprehensive assignment of the experimental bands to the vibrational normal modes using the potential energy distribution (PED) method. Small energy discrepancies were successfully explained using larger, three-molecule models that included some of the intermolecular interactions identified in the crystal. In general, the DFT models based on the atomic positions from the XRD experiments allowed for a much better agreement between the DFT-calculated and experimental vibrational spectra than the models obtained without this experimental input.

The approach we used elegantly demonstrates that an intelligent design of computational models, based on the skilful use of crystallographic data, allows for better representation of structural features in the condensed phase resulting from the interaction of intra- and intermolecular factors, and thus for improved DFT-assisted assignment of vibrational spectra.

## Figures and Tables

**Table 1:** DKP ring conformation in crystal structures of various cyclic dipeptides. Conformation in italics: assignment contested.

Peptide	Ring Conformation (from the Reference in Parentheses)	Deviation from the Mean Plane (Å)
Cyclo(l-Cys-d-Cys)	Planar (this work)	0.003
Cyclo(Gly-Gly)	Planar [63]	0.13
Cyclo(Gly-Gly)	Planar [64,65]	Not stated
Cyclo(Gly-Gly)	Planar [19]	0.009
Cyclo(l-homoCys-l-homoCys)	Planar [67]	0.0656–0.0668
Cyclo(l-Ser-l-Ser)	Planar [66]	0.020
Cyclo(l-His-l-Asp) (trihydrate)	Planar [68]	<0.02
Cyclo(l-Met-Gly)	Planar [69]	Not stated
Cyclo(l-Ser-l-His) (monohydrate)	Nearly planar [70]	0.018
Cyclo(l-Ser-l-Tyr)	Nearly planar [71]	Not stated
Cyclo(l-His-d-His)	Nearly planar (chair) [24]	Not stated
Cyclo(d-Ala-l-Ala)	Nearly planar (chair) [20]	Not stated
Cyclo(d-Ala-l-Ala)	Nearly planar [25]	0.008
Cyclo(d-Ala-l-Ala)	Nearly planar [72]	0.016
Cyclo(l-Trp-l-Trp) (cocrystal with DMSO)	Nearly planar [73]	Not stated
Cyclo(l-Ala-l-Asp)	Nearly planar (flagpole boat) [74]	Not stated
Cyclo(l-Thr-l-His) (dihyrate)	Flagpole boat [75]	0.063
Cyclo(Gly-l-Tyr)	Flagpole boat [71]	Not stated
Cyclo(l-Leu-l-His)	Flagpole boat [76]	Not stated
Cyclo(l-Leu-l-Tyr) (monohydrate)	Boat [77]	Not stated
Cyclo(l-Pro-l-Phe)	Boat [78]	Not stated
Cyclo(l-Pro-d-Phe)	Boat [79]	Not stated
Cyclo(l-Pro-d-Phe)	Boat [78]	Not stated
Cyclo(l-Met-l-Met)	Boat [80]	Not stated
Cyclo(l-Met-l-Met)	Boat [81]	Not stated
Cyclo(l-Met-l-Met)	*Twist boat* [82]	Not stated
Cyclo(l-Glu-l-Glu)	Boat [83]	Not stated
Cyclo(l-Pro-Gly)	Boat [84]	Not stated
Cyclo(l-Pro-l-Pro)	Boat [81]	Not stated
Cyclo(l-Pro-l-Leu)	Boat [85]	Not stated
Cyclo(l-Phe-l-Pro)	Boat [86]	Not stated
Cyclo(l-Asp-l-Asp)	Boat [87]	Not stated
Cyclo(l-Asp-l-Asp)	Boat [88]	Not stated
Cyclo(l-Trp-l-Pro)	Boat [73]	Not stated
Cyclo(l-Ala-l-Ala)	Bowsprit [89]	Not stated
Cyclo(l-Ala-l-Ala)	Twist boat [20]	Not stated
Cyclo(l-Tyr-l-Pro)	Flattened chair [21]	Not stated
rac-cyclo(d-Pro-l-Tyr/l-Pro-d-Tyr)	Twist boat [90]	Not stated
Cyclo-l-cystine	Twist boat [46]	Not stated

**Table 2:** Summary of the PED-based assignments of the main bands in the experimental vibrational spectra of solid cyclo(l-Cys-d-Cys).

Vibration	Experimental Wavenumbers (cm^−1^)	
	IR (Antisymmetric Vibrations)	Raman (Symmetric Vibrations)
N-H stretching	3185	3160
C-H stretching (methylene)	2970, 3040	2944, 2988
C-H stretching (DKP ring)	2886	2894
S-H stretching	2547	2549
C=O stretching	1667	1664
Ring in-plane deformations + methylene group scissoring	1462, 1484	1435, 1517
Ring in-plane deformations + HCN bending	1325	1300
Hydrogen wagging	1244, 1295	1250, 1300
C-H twisting (methylene group) + HCN bending	1190	1201
N-C stretching	1100	1137
C_ring_-C_methylene_ stretching	1034	1007
Methylene hydrogens rocking + CSH bending	814, 967	785, 976
Out-of-plane ring deformations	707, 772, 912	679, 862
Delocalised S-C and C-C stretching + out-of-plane N-H bending	672	654
OCN bending	431	

## Data Availability

Data will be made available upon reasonable request.

## Supplementary Materials

The following supporting information can be downloaded at: https://www.mdpi.com/article/10.3390/molecules28155902/s1, Table S1. Crystal data and structure refinement for investigated compound: cyclo(L-Cys-D-Cys); Table S2. Bond lengths for investigated compound: cyclo(L-Cys-D-Cys); Table S3. Valence angles for investigated compound: cyclo(L-Cys-D-Cys); Table S4. Torsion angles for investigated compound: cyclo(L-Cys-D-Cys); Table S5. The geometry of hydrogen bonds in the crystal of investigated compound: cyclo(L-Cys-D-Cys); Table S6. The values of the energies (kJ/mol) calculated for selected pairs of adjacent molecules in the crystal of investigated compound: cyclo(L-Cys-D-Cys); Figure S1. Structure of the cyclo(L-Cys-D-Cys) single molecule model optimized at B3LYP/Aug-cc-pVDZ level of theory (green) superimposed onto the structure derived from the XRD experiment in the solid state (red); Figure S2. Structure of the cyclo(L-Cys-D-Cys) three molecules model (green) superimposed onto the structures derived from the XRD experiment in the solid state (red). Molecules are involved in (a) N−H···O, (b) S−H···O hydrogen bonding; Table S7. Energy of the cyclo(L-Cys-D-Cys) conformers optimized at the B3LYP/Aug-cc-pVDZ level of theory; Figure S3. Experimental (dark-colored lines) and theoretical (light-colored lines) IR spectra of cyclo(L-Cys-D-Cys) in the full wavenumber range. Theoretical spectra were calculated using a one-molecule in vacuum model at B3LYP/Aug-cc-pVDZ level of theory; Figure S4. Experimental (dark-colored lines) and theoretical (light-colored lines) Raman spectra of cyclo(L-Cys-D-Cys) in the full wavenumber range. Theoretical spectra were calculated using a one-molecule in vacuum model at B3LYP/Aug-cc-pVDZ level of theory. ‘Diff. grating’ refers to diffraction grating used during Raman measurements; Table S8. PED analysis for the one-molecule model of cyclo(L-Cys-D-Cys) at B3LYP/Aug-cc-pVDZ level of theory; atom coordinates and energies of the computational systems.
