# Supplementary material for: The Structural Characterisation and DFT-Aided Interpretation of Vibrational Spectra for Cyclo(l-Cys-d-Cys) Cyclic Dipeptide in a Solid State
Source: Molecules. 2023 Aug 5;28(15):5902. doi: 10.3390/molecules28155902 (PMC10421304; doi:10.3390/molecules28155902)
Supplement: Supplementary file 1 [file molecules-28-05902-s001.zip › molecules-2509760-supplementary.pdf]

Supplementary Materials

S1.Results

S1.1 Crystallographic analysis

Table S1. Crystal data and structure refinement for investigated compound: cyclo(L-Cys-D-Cys).

Empirical formula	$C_6H_{10}N_2O_2S_2$
Formula weight/gmol^{-1}	206.28
Temperature/K	100(2)
Crystal system	triclinic
Space group	*P*-1
a/Å	5.3000(7)
b/Å	5.5277(7)
c/Å	8.6655(11)
α/°	104.423(11)
β/°	98.295(10)
γ/°	111.190(12)
Volume/Å^3	221.39(5)
Z	1
ρ_{calc}/g cm^{-3}	1.548
μ/mm^{-1}	0.562
F(000)	108.0
Crystal size/mm^3	0.10 × 0.08 × 0.03
Radiation	Mo*K*α (λ = 0.71073)
2*Θ* range for data collection/°	5.026 to 52.734
Index ranges	$-5 \leq h \leq 6$, $-6 \leq k \leq 6$, $-10 \leq l \leq 9$
Reflections collected	1566
Independent reflections	896 [R_{int} = 0.0312, R_{sigma} = 0.0630]
Data/restraints/parameters	896/1/61
Goodness-of-fit on F^2	1.080
Final *R* indexes [I>=2σ (I)]	R_1 = 0.0430, wR_2 = 0.0826
Final *R* indexes [all data]	R_1 = 0.0561, wR_2 = 0.0886
Largest diff. peak/hole / e Å^{-3}	0.38/-0.34

Table S2. Bond lengths for investigated compound: cyclo(L-Cys-D-Cys).

Atom	Atom	Length (XRD)/Å	Length (DFT)/Å	Atom	Atom	Length (XRD)/Å	Length (DFT)/Å
C1	C2	1.511(4)	1.528	C2	N5	1.459(3)	1.456
C1	N5[1]	1.333(3)	1.357	C3	S6	1.816(3)	1.842
C1	O4	1.235(3)	1.226	N5	C1[1]	1.333(3)	1.357
C2	C3	1.527(4)	1.539				

[1]1-X,-Y,1-Z

Table S3. Valence angles for investigated compound: cyclo(L-Cys-D-Cys).

Atom	Atom	Atom	Angle (XRD)/°	Angle (DFT)/°	Atom	Atom	Atom	Angle (XRD)/°	Angle (DFT)/°
N5[1]	C1	C2	119.1(2)	117.8	N5	C2	C1	113.7(2)	113.8
O4	C1	C2	118.6(2)	119.6	N5	C2	C3	111.0(2)	111.9
O4	C1	N5[1]	122.4(2)	122.6	C2	C3	S6	114.04(17)	114.9
C1	C2	C3	109.9(2)	110.1	C1[1]	N5	C2	127.2(2)	128.4

[1]1-X,-Y,1-Z

Table S4. Torsion angles for investigated compound: cyclo(L-Cys-D-Cys).

A	B	C	D	Angle (XRD)/°	Angle/° (DFT)	A	B	C	D	Angle (XRD)/°	Angle/° (DFT)
C1	C2	C3	S6	-56.2(2)	-63.7	N5[1]	C1	C2	N5	-0.8(4)	-1.1
C1	C2	N5	C1[1]	0.9(4)	1.3	N5	C2	C3	S6	70.5(2)	63.9
C3	C2	N5	C1[1]	-123.6(3)	-126.9	O4	C1	C2	C3	-54.7(3)	-53.1
N5[1]	C1	C2	C3	124.3(3)	-127.6	O4	C1	C2	N5	-179.8(2)	-179.6

[1]1-X,-Y,1-Z

Table S5. The geometry of hydrogen bonds in the crystal of investigated compound: cyclo(L-Cys-DCys).

D–H	A	d(D–H)	d(H···A)	d(D···A) (Å)	< D–H···A (°)
N1–H1	O5[ii]	0.87(3)<br>*1.033*	1.99(3)<br>*1.817*	2.843(3)<br>*2.850*	168(3)<br>*178.6*
S6–H6	O5[iii]	1.22(3)	2.32(3)	3.387(2)	144(2)

Symmetry codes: (ii) $x - 1$, $y - 1$, z; (iii) x, $-y + 1$, z. Values in *italics* were DFT-calculated using B3LYP/Aug-cc-pVDZ level of theory.

Table S6. The values of the energies (kJ/mol) calculated for selected pairs of adjacent molecules in the crystal of investigated compound: cyclo(L-Cys-D-Cys).

N	Symmetry operation	R	Intermolecular interactions identified within the pair of molecules	E_{ele}	E_{pol}	E_{dis}	E_{rep}	E_{tot}
1	$x - 1, y - 1, z$	6.12	N–H···O	-90.6	-19.9	-18.4	89.4	-71.4
2	$x, -y + 1, z$	5.53	S–H···O	-29.8	-9.0	-28.4	36.6	-40.3
3	$-x + 2, -y, -z + 2$	9.48	S···S	-7.0	-0.4	-4.7	8.9	-6.3
4	$-x + 2, -y +1, -z + 2$	8.69	S···S	-0.5	-1.0	-10.4	11.7	-3.0
5	$-x, -y, z - 1$	5.3	H···H	-5.7	-5.5	-26.5	23	-19.0
6	$x, y - 1, z - 1$	9.04	H···H	-1.8	-0.8	-7.1	3.7	-6.3
7	$x - 1, y - 1, z - 1$	8.66	C–H ···S	-13	-1.7	-10.2	14.8	-14.7

Energy of the interactions are defined as follows: E_{ele}, E_{pol}, E_{dis}, E_{rep}, are electrostatic, polarization, dispersion and exchange-repulsion components to the total energy, E_{tot}.

S1.2 Optimizing the model of single cyclo(L-Cys-D-Cys) molecule

Optimizing the geometry of a single molecule of cyclo(L-Cys-D-Cys) at the B3LYP/Aug-cc-pVDZ level of theory (without imposing the C_i symmetry) led to a saddle point with one imaginary vibrational frequency (both at *Tight* and *VeryTight* convergence criteria), presumably due to the model lacking the intermolecular hydrogen bonds present in the crystal system. Similar results were obtained for the B3LYP/cc-pVDZ level of theory. Curiously, augmenting the basis set in this model with diffuse functions (B3LYP/May-cc-pVDZ and B3LYP/Jul-cc-pVDZ levels of theory) led to all frequencies being positive and the mutual exclusion rule being obeyed, but the problem with imaginary frequencies reappeared for augmentations with a higher number of diffuse functions (Jul-cc-pVDZ and Aug-cc-pVDZ levels of theory).

Alternative ways to obtain a valid system were thus considered. Imposing a perfect C_i symmetry in a single molecule model proved ineffective by itself, as again imaginary frequencies were obtained for all Dunning-type double-zeta basis sets tested (cc-pVDZ, May-cc-pVDZ, Jun-cc-pVDZ, Jul-cc-pVDZ and Aug-cc-pVDZ). The effect of GD3 empirical dispersion was then investigated; however, its incorporation alone proved ineffective in the cc-pVDZ, May-cc-pVDZ and Jul-cc-pVDZ basis sets. Optimization at the B3LYP/Jul-cc-pVDZ+GD3 level of theory converged to a minimum after the convergence criteria were changed to *VeryTight*, but the resulting model did not respect the rule of mutual exclusion, even though the geometry was not far from the perfect C_i symmetry. The same problem occurred for optimization at B3LYP/Aug-cc-pVDZ+GD3 level of theory at *Tight* convergence criteria.

Finally, a proper model was obtained using a procedure described in the main text of the manuscript (see Chapter 2.2.2).

S1.3 Structure of the optimized model

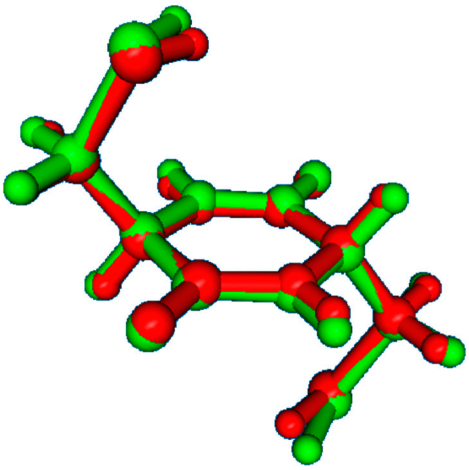

Figure S1. Structure of the cyclo(L-Cys-D-Cys) single molecule model optimized at B3LYP/Aug-cc-pVDZ level of theory (green) superimposed onto the structure derived from the XRD experiment in the solid state (red)

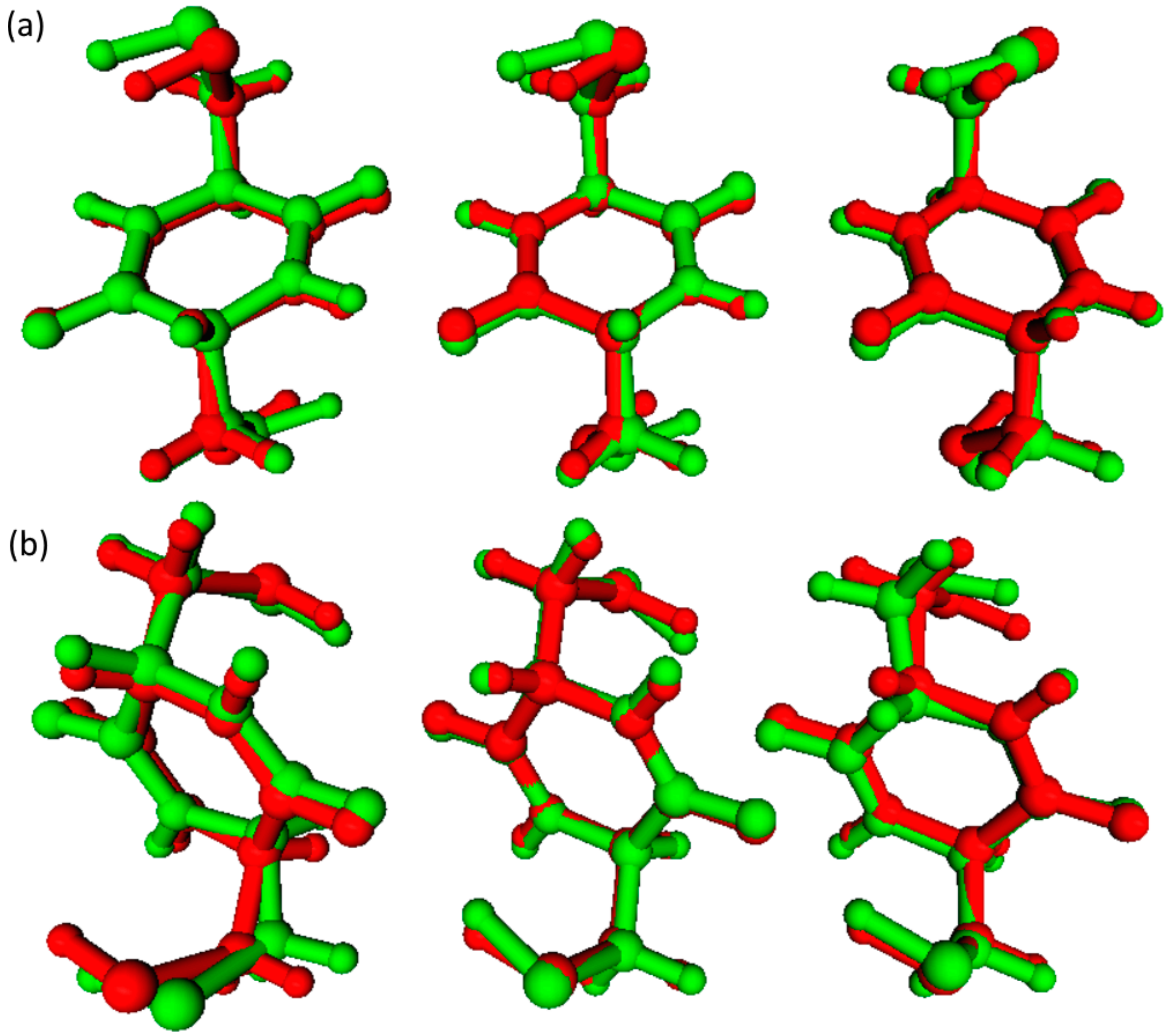

Figure S2. Structure of the cyclo(L-Cys-D-Cys) three molecules model (green) superimposed onto the structures derived from the XRD experiment in the solid state (red). Molecules are involved in (a) N−H···O, (b) S−H···O hydrogen bonding

S1.4 Conformational analysis

Table S7. *Energy of the cyclo(L-Cys-D-Cys) conformers optimized at the B3LYP/Aug-cc-pVDZ level of theory.*

#	Source of the initial geometry	Relative energy [kJ/mol]
1	Conf8	0.00
2	Conf6, Conf7	0.58
3	Conf5	2.45
4	XRD data	4.00
5	Conf9	14.64
6	Conf2	16.29
7	Conf4	18.34
8	Conf3	18.47
9	Conf1	30.04

S1.5 Vibrational spectroscopy

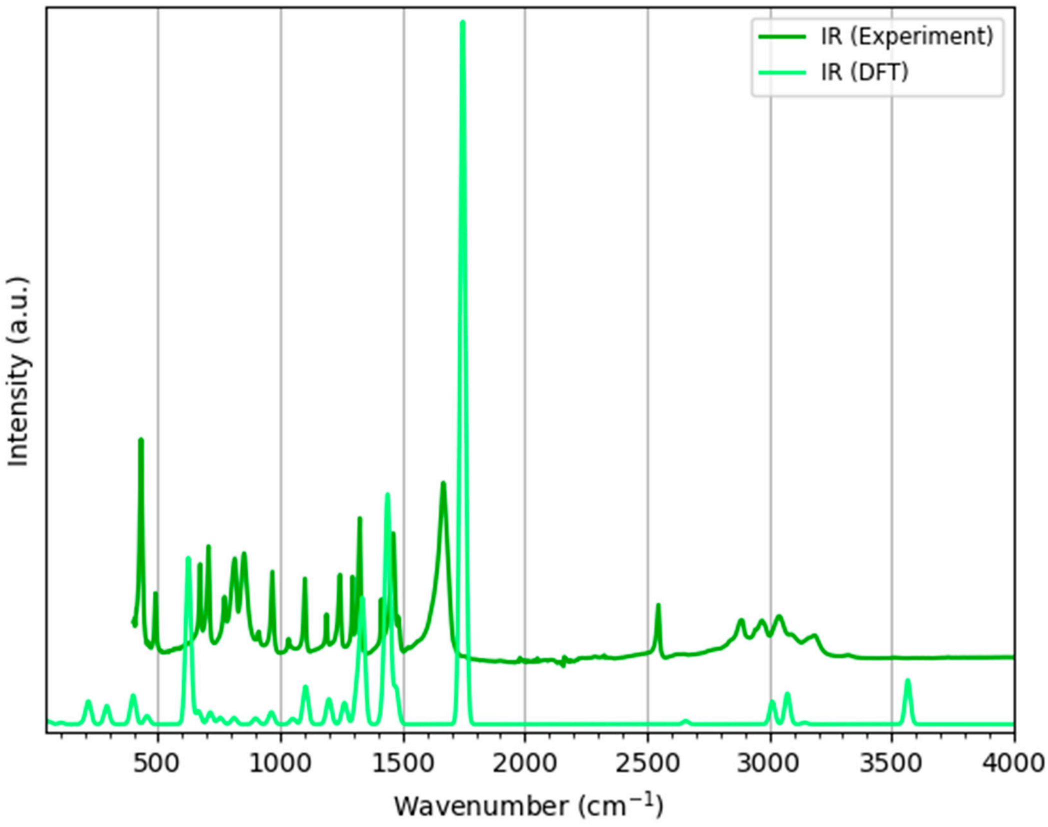

Figure S3. Experimental (dark-colored lines) and theoretical (light-colored lines) IR spectra of cyclo(L-Cys-D-Cys) in the full wavenumber range. Theoretical spectra were calculated using a one-molecule in vacuum model at B3LYP/Aug-cc-pVDZ level of theory.

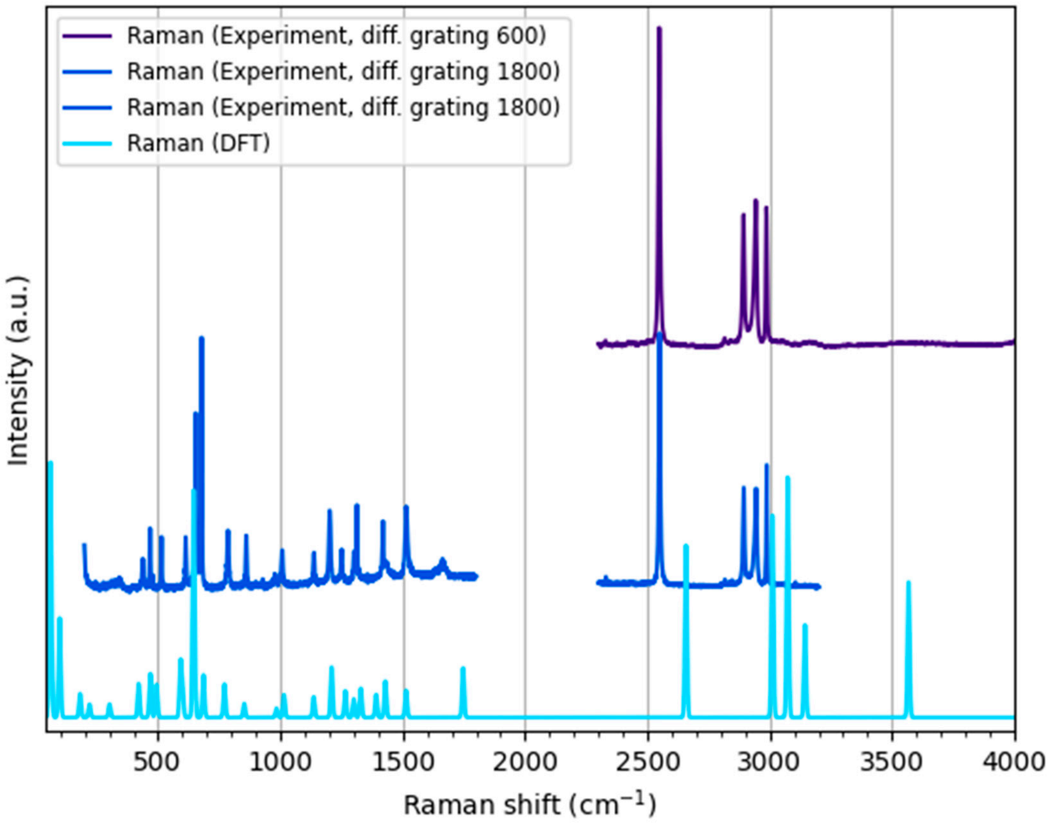

Figure S4. Experimental (dark-colored lines) and theoretical (light-colored lines) Raman spectra of cyclo(L-Cys-D-Cys) in the full wavenumber range. Theoretical spectra were calculated using a one-molecule in vacuum model at B3LYP/Aug-cc-pVDZ level of theory. 'Diff. grating' refers to diffraction grating used during Raman measurements.

Table S8. PED analysis for the one-molecule model of cyclo(L-Cys-D-Cys) at B3LYP/Aug-cc-pVDZ level of theory

**Cyclo(L-Cys-D-Cys)**							
**Mode No**	**Theoretical**					**Experimental**	
	**Wavenumber**	**IR Intensity**	**Raman activity**[a]	**Raman Int. 532.0 nm**[b]	**Assignment with PED (≥10%)**[c] *In italics: tentative assignments outside PED method*	**IR**	**Raman**
1	4	0.2	0.0	0.0	$\tau C1N5^{i}C2^{i}C1^{i}$(-24), $\tau N5^{i}C2^{i}C1^{i}N5$(28), $\tau C2^{i}C1^{i}N5C2$(29)		
**2**	52	0.4	0.0	0.0	$\tau C1N5^{i}C2^{i}C1^{i}$(14), $\tau N5^{i}C2^{i}C1^{i}N5$(15), $\tau C2^{i}C1^{i}N5C2$(-12), $\tau S6C3C2N5$(-16), $\tau S6^{i}C3^{i}C2^{i}N5^{i}$(-18)		
**3**	60	0.0	1.0	106.6	$\tau C1N5^{i}C2^{i}C1^{i}$(-22), $\tau N5^{i}C2^{i}C1^{i}N5$(15), $\tau C2^{i}C1^{i}N5C2$(-22)		
**4**	98	0.0	0.7	41.4	$\tau S6C3C2N5$(22), $\tau S6^{i}C3^{i}C2^{i}N5^{i}$(-24)		
**5**	103	0.2	0.0	0.0	$\tau N5^{i}C2^{i}C1^{i}N5$(15), $\tau S6C3C2N5$(14), $\tau S6^{i}C3^{i}C2^{i}N5^{i}$(15)		
**6**	181	0.0	0.3	9.9	$\delta S6C3C2$(21), $\delta S6^{i}C3^{i}C2^{i}$(20)		
**7**	195	0.2	0.0	0.0	$\delta S6C3C2$(-25), $\delta S6^{i}C3^{i}C2^{i}$(23), $\Gamma C3^{i}N5^{i}C1^{i}C2^{i}$(12), $\Gamma C3N5C1C2$(12)		
**8**	215	3.2	0.0	0.0	$\tau H6S6C3C2$(32), $\tau H6^{i}S6^{i}C3^{i}C2^{i}$(39)		

**9**	220	0.0	0.2	5.4	τH6S6C3C2(-37), τH6^{i}S6^{i}C3^{i}C2^{i}(46)		
**10**	290	2.6	0.0	0.0	δC3C2C1(-20), δC3^{i}C2^{i}C1^{i}(20)		
**11**	302	0.0	0.3	5.5	δC3C2C1(20), δC3^{i}C2^{i}C1^{i}(20)		340 vvw
**12**	398	4.1	0.0	0.0	δO4C1N5^{i}(-30), δO4^{i}C1^{i}N5(30)	431 vs	
**13**	420	0.0	1.0	14.0	δN5^{i}C2^{i}C1^{i}(28), δC2^{i}C1^{i}N5(-15)		439 vw
**14**	455	1.2	0.0	0.0	τH5^{i}N5^{i}C1C2(-14), τH5N5C1^{i}C2^{i}(-14), ΓC3^{i}N5^{i}C1^{i}C2^{i}(-10), ΓC3N5C1C2(-10)	490 m	
**15**	468	0.0	1.5	18.2	δC1N5^{i}C2^{i}(27), δC2N5C1^{i}(13)		469 m
							481 w
**16**	494	0.0	1.2	13.7			516 w
**17**	592	0.0	2.4	22.3	τH5^{i}N5^{i}C1C2(25), τH5N5C1^{i}C2^{i}(-25)		614 w
**18**	600	0.0	1.2	10.6	νC2^{i}C1^{i}(28), δO4C1N5^{i}(-12), δO4^{i}C1^{i}N5(-12)		
**19**	624	23.6	0.0	0.0	νS6C3(-12), νS6^{i}C3^{i}(12), τH5^{i}N5^{i}C1C2(28), τH5N5C1^{i}C2^{i}(28)		
**20**	645	0.0	11.2	94.6	νC2^{i}C1^{i}(15), νS6C3(24), νS6^{i}C3^{i}(24)		654 vs
**21**	665	1.9	0.0	0.0	νS6C3(24), νS6^{i}C3^{i}(-24)	672 m	

**22**	685	0.0	2.2	17.6	ΓO4^{i}C2^{i}N5C1^{i}(15), ΓO4C2N5^{i}C1(-15)		679 vs
**23**	713	1.7	0.0	0.0	ΓO4^{i}C2^{i}N5C1^{i}(-14), ΓO4C2N5^{i}C1(-14)	707 m	
**24**	754	0.9	0.0	0.0	τH3BC3C2N5(11), τH3A^{i}C3^{i}C2^{i}N5^{i}(12)	772 w	
**25**	771	0.0	2.0	13.8	δH6S6C3(19), δH6^{i}S6^{i}C3^{i}(19), τH3BC3C2N5(-14)		785 m
**26**	810	0.9	0.0	0.0	δH6S6C3(-10), δH6^{i}S6^{i}C3^{i}(10), δC2^{i}C1^{i}N5(18)	814 m	
**27**	852	0.0	0.9	5.8	ΓO4^{i}C2^{i}N5C1^{i}(10), ΓO4C2N5^{i}C1(-10), ΓC3^{i}N5^{i}C1^{i}C2^{i}(-11), ΓC3N5C1C2(11)		862 m
					431 overtone	852 m	
**28**	899	0.9	0.0	0.0		912 vw	
**29**	963	1.7	0.0	0.0	δH6S6C3(16), δH6^{i}S6^{i}C3^{i}(-16)	967 m	
**30**	984	0.0	0.7	3.8	δH6S6C3(22), δH6^{i}S6^{i}C3^{i}(22)		976 vw
**31**	1014	0.0	1.9	9.4	νC3C2(26), νC3^{i}C2^{i}(26)		1007 w
**32**	1051	0.8	0.0	0.0	νC3C2(-12), νC3^{i}C2^{i}(12), δC2N5C1^{i}(11)	1034 vw	
**33**	1103	5.4	0.0	0.0	νN5^{i}C2^{i}(-18), νN5C2(18)	1100 m	
**34**	1136	0.0	2.0	8.5	νN5^{i}C2^{i}(-10), νN5C2(-12), δH3A^{i}C3^{i}S6^{i}(10)		1137 w

**35**	1199	3.6	0.0	0.0	δH2C2N5(10), δH2^{i}C2^{i}C1^{i}(15), δH3BC3S6(-11), δH3A^{i}C3^{i}S6^{i}(-12), τH3B^{i}C3^{i}C2^{i}N5^{i}(19)	1190 w	
**36**	1209	0.0	5.2	20.8	δH2^{i}C2^{i}C1^{i}(21), δH3BC3S6(13), τH3B^{i}C3^{i}C2^{i}N5^{i}(16)		1201 s
**37**	1262	3.1	0.0	0.0	δH3BC3S6(10), δH3A^{i}C3^{i}S6^{i}(-15), τH2C2C1N5^{i}(-12), τH2^{i}C2^{i}C1^{i}N5(-14), τH3AC3S6H6(17)	1244 m	
**38**	1265	0.0	2.9	11.1	δH3A^{i}C3^{i}S6^{i}(20), τH2^{i}C2^{i}C1^{i}N5(11), τH3AC3S6H6(20)		1250 w
**39**	1300	0.0	2.1	7.6	νN5^{i}C1(13), δH2C2N5(19), δH2^{i}C2^{i}C1^{i}(-16)		1300 w
**40**	1313	4.9	0.0	0.0	δH2^{i}C2^{i}C1^{i}(11), δH3BC3S6(13), τH2C2C1N5^{i}(15), τH3B^{i}C3^{i}C2^{i}N5^{i}(-10)	1295 m	
**41**	1329	0.0	3.4	12.1	δH3BC3S6(-12), τH2C2C1N5^{i}(-21), τH2^{i}C2^{i}C1^{i}N5(17)		1312 m
**42**	1335	17.4	0.0	0.0	δH2C2N5(20), τH2^{i}C2^{i}C1^{i}N5(-17)	1326 s	
**43**	1391	0.0	2.9	9.5	νC2^{i}C1^{i}(10), δH5^{i}N5^{i}C1(-17), δH5N5C1^{i}(-17)		
**44**	1422	2.7	0.0	0.0	δH5^{i}N5^{i}C1(10), δH5N5C1^{i}(-10), δH3AC3H3B(27), δH3B^{i}C3^{i}H3A^{i}(-18)	1413 m	
**45**	1429	0.0	4.7	15.1	δH3AC3H3B(43), δH3B^{i}C3^{i}H3A^{i}(43)		1421 m
							1435 w
**46**	1439	31.9	0.0	0.0	νN5^{i}C1(-17), νN5C1^{i}(18), δH3AC3H3B(11), δH3B^{i}C3^{i}H3A^{i}(-18)	1462 s	
**47**	1473	5.1	0.0	0.0	δH5^{i}N5^{i}C1(-22), δH5N5C1^{i}(22)	1484 w	
**48**	1514	0.0	3.8	11.2	νN5^{i}C1(-15), νC2^{i}C1^{i}(10), δH5^{i}N5^{i}C1(12), δH5N5C1^{i}(12)		1517 s

					431+1101 combination band	1523 vvw	
**49**	1746	100.0	0.0	0.0	νO4C1(-37), νO4^{i}C1^{i}(37)	1667 vs	
**50**	1747	0.0	8.5	20.6	νO4C1(38), νO4^{i}C1^{i}(38)		1664 w b
					672+1667 combination band	2325 vw	
					Nitrogen		2330 vw
**51**	2658	0.5	0.0	0.0	νS6H6(-50), νS6^{i}H6^{i}(50)	2547 m	
**52**	2658	0.0	55.7	71.5	νS6H6(50), νS6^{i}H6^{i}(50)		2549 vs
					1421 overtone		2816 vw
							2846 vw
**53**	3011	0.0	81.1	84.1	νC2H2(49), νC2^{i}H2^{i}(49)		2894 m
**54**	3012	3.2	0.0	0.0	νC2H2(-49), νC2^{i}H2^{i}(49)	2886 w	
						2944 vw	
**55**	3073	4.4	0.0	0.0	νC3H3A(-11), νC3H3B(-39), νC3^{i}H3A^{i}(11), νC3^{i}H3B^{i}(39)	2970 w	
**56**	3074	0.0	100.0	100.0	νC3H3A(11), νC3H3B(39), νC3^{i}H3A^{i}(11), νC3^{i}H3B^{i}(39)		2944 m
**57**	3144	0.0	40.0	38.4	νC3H3A(39), νC3H3B(-11), νC3^{i}H3A^{i}(39), νC3^{i}H3B^{i}(-11)		2988 m
**58**	3144	0.2	0.0	0.0	νC3H3A(-39), νC3H3B(11), νC3^{i}H3A^{i}(39), νC3^{i}H3B^{i}(-11)	3040 w	
						3090 w	
**59**	3566	6.3	0.0	0.0	νN5^{i}H5^{i}(-50), νN5H5(50)	3185 w	

**60**	3568	0.0	74.1	56.2	νN5^{i}H5^{i}(-50), νN5H5(-50)	3160 vw b

[a] The Raman activity values were normalized so that the largest activity equals 100. Bands below 250 cm^{-1} were not taken into account in the normalization procedure.

[b] The Raman intensity values were calculated for the specified excitation laser line wavelength. They were normalized so that the largest intensity equals 100. Bands below 250 cm^{-1} were not taken into account in the normalization procedure.

[c] The assignment of vibration type was performed using the VEDA4 software. Atom numbering is presented in Figure 1. The numbers in parentheses denote the percentage contribution to the potential energy for the given oscillation in a mode and the negative numbers denote oscillations in counterphase. The modes contributing less than 10% to the energy of a vibration were not included; ν denotes stretching modes, δ – bending modes, τ – torsion modes and Γ – out-of-plane torsion modes.

S2. Atom coordinates for conformational analysis

S2.1 Optimized one- and three-molecule models discussed in the paper

One-molecule model optimized at B3LYP/Aug-cc-pVDZ level of theory, energy - 1291.12983129 a.u.

```
H 1.8047190000 -1.0373580000 -0.9171690000
H 1.8793560000 1.4082200000 -0.4416310000
H 1.8718340000 0.4368390000 -2.8808300000
S 0.4910180000 -0.9732100000 3.1649290000
H 0.1052760000 -2.1301090000 2.5707480000
N 1.0717870000 0.8160840000 -0.2801870000
C 0.9354360000 -0.3819610000 -1.0965110000
H 0.8530960000 -0.9935400000 -3.1561540000
C 0.9311010000 -0.0531610000 -2.6003140000
O 0.5028490000 2.2647410000 1.3666690000
C 0.2819670000 1.2343920000 0.7408910000
H -1.8047190000 1.0373580000 0.9171690000
H -1.8793560000 -1.4082200000 0.4416310000
H -1.8718340000 -0.4368390000 2.8808300000
S -0.4910180000 0.9732100000 -3.1649290000
H -0.1052760000 2.1301090000 -2.5707480000
N -1.0717870000 -0.8160840000 0.2801870000
C -0.9354360000 0.3819610000 1.0965110000
H -0.8530960000 0.9935400000 3.1561540000
C -0.9311010000 0.0531610000 2.6003140000
O -0.5028490000 -2.2647410000 -1.3666690000
C -0.2819670000 -1.2343920000 -0.7408910000
```

Three-molecule model incorporating the-N-H···O-C- hydrogen bonds optimized at B3LYP/Aug-cc-pVDZ level of theory, energy -3873.43988626 a.u.

```
H 5.0522940000 4.3603890000 -0.9394650000
H 4.7643420000 6.8564940000 -0.8321980000
H 4.9269180000 5.5880680000 -3.0680380000
S 4.2510590000 4.4170710000 2.9032770000
H 3.8767370000 3.1750050000 2.5072840000
N 4.0702860000 6.1733980000 -0.5483790000
C 4.1045740000 4.8684860000 -1.1922260000
H 4.1417470000 3.9981640000 -3.1590530000
C 4.0672610000 4.9997180000 -2.7238180000
O 3.4849170000 7.6353880000 1.0816200000
C 3.3567910000 6.5395140000 0.5487970000
C 3.0086040000 3.9273860000 -0.7014400000
O 2.8863080000 2.8250060000 -1.2632920000
C 2.6348600000 5.2461490000 2.5842600000
H 2.6775860000 6.2003790000 3.1196600000
C 2.3466110000 5.5374650000 1.0987540000
N 2.2355520000 4.3114000000 0.3213680000
H 2.6808660000 6.9696620000 -2.9166320000
S 2.5143720000 5.7165880000 -3.4083570000
H 1.8271810000 4.6400310000 3.0090150000
H 1.5222190000 3.6279150000 0.6264830000
H 1.8454040000 -0.8842400000 -0.9652900000
H 1.3749920000 6.0565210000 1.0627880000
H 1.6641090000 1.5978990000 -0.7150260000
H 1.6219810000 0.4648700000 -3.0014700000
S 0.7979000000 -0.6831480000 3.1806250000
H 0.4880250000 -1.9346570000 2.7600420000
```

```
N 0.9498390000 0.9167540000 -0.4088620000
C 0.8944950000 -0.3477900000 -1.1259310000
H 0.7866970000 -1.0995270000 -3.1409990000
C 0.7701160000 -0.1242380000 -2.6445110000
O 0.3095800000 2.3994690000 1.1821250000
C 0.1865480000 1.2951530000 0.6241620000
H -5.0522940000 -4.3603890000 0.9394650000
H -4.7643420000 -6.8564940000 0.8321980000
H -4.9269180000 -5.5880680000 3.0680380000
S -4.2510590000 -4.4170710000 -2.9032770000
H -3.8767370000 -3.1750050000 -2.5072840000
N -4.0702860000 -6.1733980000 0.5483790000
C -4.1045740000 -4.8684860000 1.1922260000
H -4.1417470000 -3.9981640000 3.1590530000
C -4.0672610000 -4.9997180000 2.7238180000
O -3.4849170000 -7.6353880000 -1.0816200000
C -3.3567910000 -6.5395140000 -0.5487970000
C -3.0086040000 -3.9273860000 0.7014400000
O -2.8863080000 -2.8250060000 1.2632920000
C -2.6348600000 -5.2461490000 -2.5842600000
H -2.6775860000 -6.2003790000 -3.1196600000
C -2.3466110000 -5.5374650000 -1.0987540000
N -2.2355520000 -4.3114000000 -0.3213680000
H -2.6808660000 -6.9696620000 2.9166320000
S -2.5143720000 -5.7165880000 3.4083570000
H -1.8271810000 -4.6400310000 -3.0090150000
H -1.5222190000 -3.6279150000 -0.6264830000
H -1.8454040000 0.8842400000 0.9652900000
H -1.3749920000 -6.0565210000 -1.0627880000
H -1.6641090000 -1.5978990000 0.7150260000
H -1.6219810000 -0.4648700000 3.0014700000
S -0.7979000000 0.6831480000 -3.1806250000
H -0.4880250000 1.9346570000 -2.7600420000
N -0.9498390000 -0.9167540000 0.4088620000
C -0.8944950000 0.3477900000 1.1259310000
H -0.7866970000 1.0995270000 3.1409990000
C -0.7701160000 0.1242380000 2.6445110000
O -0.3095800000 -2.3994690000 -1.1821250000
C -0.1865480000 -1.2951530000 -0.6241620000
```

Three-molecule model incorporating the-S-H···O-C- hydrogen bonds optimized at B3LYP/Aug-cc-pVDZ+GD3 level of theory, energy -3873.50827512 a.u.

```
H -4.0182450000 5.8550640000 1.1906410000
H -3.6705830000 3.4647000000 0.8847930000
H -3.2685410000 4.6186680000 3.2143280000
S -2.5129650000 5.0530640000 -3.3246890000
H -2.5417040000 6.3487690000 -2.9245290000
N -2.9953960000 4.1521820000 0.5699760000
C -2.9955470000 5.4511830000 1.2328960000
H -2.7456520000 6.3191270000 3.1849750000
C -2.6035040000 5.3380500000 2.7178660000
O -2.1947310000 2.5507510000 -0.8060710000
C -2.1447050000 3.7050430000 -0.3781060000
C -2.1216530000 6.4890280000 0.5330350000
H -1.7982040000 0.9569870000 0.9783050000
O -2.1774240000 7.6603390000 0.8844320000
H -1.6186640000 -1.5216650000 0.8624450000
H -1.4201100000 -0.3269630000 3.0837730000
C -0.9741620000 4.5919910000 -2.4196410000
H -0.7641700000 3.5573620000 -2.7089530000
C -1.0862480000 4.6743160000 -0.8908480000
N -1.3168460000 6.0515750000 -0.4684700000
H -0.7876560000 3.7639310000 2.2720440000
```

```
S -0.8403750000 4.8979850000 3.0204120000
S -1.0331950000 0.3457320000 -3.1476480000
H -1.1384040000 1.3159980000 -2.2007550000
N -0.9183440000 -0.8772930000 0.5135890000
C -0.8444580000 0.4345990000 1.1474250000
H -0.6994090000 1.2977870000 3.1145390000
C -0.6247140000 0.3025350000 2.6625620000
H -0.1402940000 5.2177310000 -2.7622730000
H -0.6918630000 6.7616300000 -0.8333310000
O -0.3632840000 -2.4833320000 -0.9769820000
H -0.1422040000 4.3031830000 -0.4581310000
C -0.2038520000 -1.3477900000 -0.5291820000
H 4.0182450000 -5.8550640000 -1.1906410000
H 3.6705830000 -3.4647000000 -0.8847930000
H 3.2685410000 -4.6186680000 -3.2143280000
S 2.5129650000 -5.0530640000 3.3246890000
H 2.5417040000 -6.3487690000 2.9245290000
N 2.9953960000 -4.1521820000 -0.5699760000
C 2.9955470000 -5.4511830000 -1.2328960000
H 2.7456520000 -6.3191270000 -3.1849750000
C 2.6035040000 -5.3380500000 -2.7178660000
O 2.1947310000 -2.5507510000 0.8060710000
C 2.1447050000 -3.7050430000 0.3781060000
C 2.1216530000 -6.4890280000 -0.5330350000
H 1.7982040000 -0.9569870000 -0.9783050000
O 2.1774240000 -7.6603390000 -0.8844320000
H 1.6186640000 1.5216650000 -0.8624450000
H 1.4201100000 0.3269630000 -3.0837730000
C 0.9741620000 -4.5919910000 2.4196410000
H 0.7641700000 -3.5573620000 2.7089530000
C 1.0862480000 -4.6743160000 0.8908480000
N 1.3168460000 -6.0515750000 0.4684700000
H 0.7876560000 -3.7639310000 -2.2720440000
S 0.8403750000 -4.8979850000 -3.0204120000
S 1.0331950000 -0.3457320000 3.1476480000
H 1.1384040000 -1.3159980000 2.2007550000
N 0.9183440000 0.8772930000 -0.5135890000
C 0.8444580000 -0.4345990000 -1.1474250000
H 0.6994090000 -1.2977870000 -3.1145390000
C 0.6247140000 -0.3025350000 -2.6625620000
H 0.1402940000 -5.2177310000 2.7622730000
H 0.6918630000 -6.7616300000 0.8333310000
O 0.3632840000 2.4833320000 0.9769820000
H 0.1422040000 -4.3031830000 0.4581310000
C 0.2038520000 1.3477900000 0.5291820000
```

S2.2 Conformers of cyclo(L-Cys-D-Cys) predicted by Avogadro software

```
Conf1
H  -2.08817 -1.13214 1.36565
C  -1.40998 -0.69717 0.62214
C  -2.10606 -0.54852 -0.73656
H  -2.88025 0.22423 -0.66926
C  -0.15398 -1.58644 0.54879
O  -0.18427 -2.81307 0.55615
S  -2.92175 -2.06518 -1.32723
H  -1.40548 -0.23051 -1.51380
H  -3.61103 -2.33714 -0.20874
N   1.04340 -0.92879 0.37948
H   1.81343 -1.55749 0.17960
H   0.74471 0.45036 -1.12867
C   1.09128 0.46155 -0.08925
C   2.51641 1.02031 0.00366
H   3.24108 0.24817 -0.28003
C   0.08985 1.29331 0.74646
O   0.17193 2.49877 0.94847
S   2.82047 2.43447 -1.10757
H   2.75402 1.32419 1.02857
H   2.54029 1.80977 -2.26018
N  -1.02928 0.60998 1.17146
H  -1.72409 1.18519 1.63300
```

```
Conf2
H  -1.90537 -1.00871 1.39622
C  -1.35709 -0.60368 0.53528
C  -2.29581 -0.68487 -0.67627
H  -2.61225 -1.72104 -0.83722
C  -0.12915 -1.51592 0.37858
O  -0.20957 -2.72926 0.21257
S  -1.61383 -0.08038 -2.25456
H  -3.19625 -0.09477 -0.47393
H  -0.81348 -1.13288 -2.48983
```

N 1.09818 -0.89541 0.39983
H 1.84918 -1.54144 0.18408
H 1.00800 0.48834 -1.12847
C 1.22952 0.49680 -0.05669
C 2.65479 1.01396 0.17982
H 3.37818 0.22222 -0.04598
C 0.17189 1.37423 0.64608
O 0.28377 2.57849 0.84953
S 3.11601 2.43005 -0.87537
H 2.80398 1.29610 1.22742
H 2.71025 1.91578 -2.04543
N -1.01385 0.75208 0.95178
H -1.72548 1.36259 1.33550

Conf3
H -2.14593 -0.97149 1.40237
C -1.44568 -0.62741 0.63194
C -2.13571 -0.56840 -0.73699
H -2.95446 0.15941 -0.69308
C -0.22663 -1.57329 0.65622
O -0.31505 -2.78991 0.78536
S -2.85679 -2.13738 -1.31055
H -1.44942 -0.22007 -1.51493
H -3.45516 -2.48520 -0.16111
N 0.99677 -0.98502 0.44117
H 1.76767 -1.63779 0.35364
H 0.79804 0.29462 -1.16471
C 1.11087 0.36090 -0.11558
C 2.54408 0.90661 -0.07755
H 2.58994 1.91085 -0.51095
C 0.11736 1.31288 0.57944
O 0.24435 2.53189 0.62068
S 3.27195 0.98006 1.59578
H 3.19416 0.25820 -0.67522
H 2.47849 1.96029 2.06047
N -1.02620 0.71519 1.06311
H -1.71397 1.35560 1.44238

Conf4
H -2.14499 -0.97030 1.40483
C -1.44439 -0.62862 0.63406
C -2.13237 -0.56476 -0.73606
H -2.93938 0.17588 -0.69841
C -0.22834 -1.57648 0.65135
O -0.31516 -2.79424 0.77029
S -2.87560 -2.12825 -1.29744
H -1.44057 -0.23445 -1.51674
H -3.47681 -2.46024 -0.14501
N 0.99823 -0.98968 0.44124
H 1.73186 -1.66646 0.26884
H 0.77354 0.27882 -1.19040
C 1.10563 0.34568 -0.14746
C 2.52754 0.91275 -0.07939
H 2.87776 0.96735 0.95732
C 0.13437 1.29782 0.58217
O 0.27493 2.51444 0.64408
S 3.71645 -0.09860 -1.02858
H 2.55699 1.92645 -0.49263
H 3.81364 -1.10817 -0.15171
N -1.01123 0.70703 1.07039
H -1.68948 1.35203 1.45897

Conf5
H -1.93930 -0.88798 1.40962
C -1.36305 -0.55803 0.53477
C -2.28132 -0.69322 -0.68808
H -2.59346 -1.73544 -0.81350
C -0.16436 -1.52325 0.46216
O -0.28895 -2.74145 0.38064
S -1.57502 -0.14495 -2.27683
H -3.18587 -0.09739 -0.52431
H -0.73897 -1.18279 -2.44139
N 1.08110 -0.94344 0.45758
H 1.83495 -1.61011 0.33710
H 1.06348 0.35703 -1.14155
C 1.26025 0.41133 -0.06568
C 2.68977 0.94250 0.10740
H 2.78008 1.95684 -0.29506
C 0.21530 1.37913 0.51792
O 0.37280 2.59412 0.59184
S 3.27969 0.96270 1.83412
H 3.38037 0.30397 -0.45457
H 2.48469 1.96335 2.24868
N -0.98881 0.81737 0.86372
H -1.69545 1.47284 1.17512

Conf6
H -1.92660 -0.91022 1.42389
C -1.37168 -0.56852 0.54107
C -2.32084 -0.67278 -0.66073
H -2.64458 -1.71016 -0.79735
C -0.18348 -1.53912 0.41638
O -0.31813 -2.75399 0.30680
S -1.65108 -0.10067 -2.25636
H -3.21615 -0.07348 -0.46292
H -0.82285 -1.13805 -2.46070
N 1.06862 -0.97151 0.40316
H 1.78623 -1.65100 0.17971
H 1.03727 0.35936 -1.19119
C 1.24980 0.38733 -0.11727
C 2.66842 0.92237 0.11075
H 2.90944 0.94947 1.17917
C 0.23061 1.34894 0.52223
O 0.40223 2.55981 0.62598
S 3.93043 -0.09750 -0.73059
H 2.76060 1.94259 -0.27633
H 3.96669 -1.08863 0.17145
N -0.97054 0.79417 0.88632
H -1.66175 1.45347 1.22442

Conf7
H -2.12260 -0.95746 1.44145
C -1.44557 -0.59130 0.66011
C -2.14410 -0.58696 -0.70439
H -1.47301 -0.23797 -1.49571
C -0.25054 -1.56077 0.60704
O -0.37498 -2.78108 0.60303
S -3.61920 0.48664 -0.71924
H -2.46006 -1.60071 -0.97278
H -2.97166 1.64550 -0.52105
N 0.98582 -0.98205 0.43847
H 1.74025 -1.64562 0.30253
H 0.79039 0.32422 -1.14485
C 1.11251 0.37346 -0.09707
C 2.55111 0.90481 -0.06677
H 2.60093 1.91560 -0.48442

C 0.13425 1.33000 0.61232
O 0.27320 2.54748 0.65837
S 3.29830 0.94346 1.59805
H 3.18734 0.26088 -0.68375
H 2.52615 1.92990 2.08501
N -1.00867 0.73877 1.09776
H -1.69174 1.37364 1.49378

Conf8
H -2.11663 -0.95516 1.44636
C -1.44321 -0.59013 0.66188
C -2.14755 -0.57457 -0.69900
H -1.47977 -0.22160 -1.49136
C -0.25513 -1.56693 0.59332
O -0.38294 -2.78687 0.58230
S -3.61962 0.50117 -0.69767
H -2.46551 -1.58612 -0.97377
H -2.96893 1.65557 -0.48484
N 0.98568 -0.99422 0.42473
H 1.70058 -1.67710 0.20271
H 0.76988 0.31044 -1.17818
C 1.10950 0.35452 -0.13609
C 2.53948 0.90030 -0.06911
H 2.89208 0.94900 0.96701
C 0.15802 1.31259 0.60892
O 0.31291 2.52708 0.67499
S 3.71031 -0.12794 -1.02191
H 2.58146 1.91402 -0.48137
H 3.83770 -1.11078 -0.11867
N -0.98642 0.73132 1.10397
H -1.65644 1.37172 1.51379

Conf9
H -2.09401 -1.07599 1.39723
C -1.43423 -0.63273 0.64163
C -2.14453 -0.54539 -0.71346
H -1.48089 -0.15185 -1.48968
C -0.21474 -1.55969 0.50337
O -0.30091 -2.77923 0.40200
S -3.62168 0.52151 -0.65580
H -2.46065 -1.54343 -1.03623
H -2.96933 1.68026 -0.47119
N 1.00618 -0.93614 0.37122
H 1.74964 -1.58361 0.13204
H 0.74804 0.46856 -1.12091
C 1.09305 0.46053 -0.08010
C 2.53416 0.97560 0.01907
H 3.23348 0.18977 -0.28854
C 0.11360 1.31787 0.75430
O 0.22310 2.52253 0.94940
S 2.87902 2.40972 -1.05408
H 2.78532 1.24324 1.05111
H 2.52236 1.84636 -2.21687
N -1.01834 0.66291 1.18190
H -1.70505 1.24888 1.64295

S2.3 Conformers of cyclo(L-Cys-D-Cys) after optimization at the B3LYP/Aug-cc-pVDZ level of theory

Conformer #1 in terms of energy, obtained from Conf8 structure, energy -1291.13135588 a.u.
H -1.777792 0.714521 -1.508662
C -1.400977 0.457668 -0.508624
C -2.549967 0.673507 0.498592
H -2.211400 0.488611 1.524681
C -0.294622 1.468583 -0.210458
O -0.529462 2.669009 -0.294714
S -4.041980 -0.355998 0.158442
H -2.878550 1.715689 0.425446
H -3.623061 -1.484477 0.784112
N 0.920207 0.981344 0.151775
H 1.604439 1.689352 0.399078
H 1.439996 -0.576899 1.457826
C 1.308197 -0.404383 0.377037
C 2.642302 -0.737890 -0.316589
H 2.547449 -0.645528 -1.404134
C 0.251997 -1.412369 -0.081124
O 0.497419 -2.613306 -0.043417
S 4.059713 0.295507 0.258151
H 2.901370 -1.774260 -0.079792
H 3.895034 1.323374 -0.611535
N -0.929971 -0.920240 -0.534000
H -1.598100 -1.626567 -0.824882

Conformer #2 in terms of energy, obtained from Conf6 structure, energy -1291.13113372 a.u.
H 1.902655 -0.133226 -2.138133
C 1.601372 0.000752 -1.086890
C 2.889532 -0.080223 -0.243036
H 3.434874 -0.992078 -0.505600
C 0.693130 -1.188535 -0.780917
O 1.098279 -2.328510 -0.983456
S 2.622829 -0.030276 1.582664
H 3.528899 0.781524 -0.473295
H 2.317963 -1.344680 1.723633
N -0.549182 -0.917718 -0.306702
H -1.085592 -1.738812 -0.043258
H -0.919188 0.377310 1.307733
C -1.033946 0.356637 0.211578
C -2.520830 0.561329 -0.120622
H -2.674253 0.612564 -1.203952
C -0.236416 1.553017 -0.310946
O -0.650936 2.694491 -0.148112
S -3.627550 -0.737878 0.586110
H -2.842172 1.511148 0.316288
H -3.572267 -1.605359 -0.455142
N 0.924001 1.283914 -0.967949
H 1.425090 2.112973 -1.268556

Conformer #3 in terms of energy, obtained from Conf5 structure, energy -1291.13042359 a.u.
H -1.204773 0.652024 -2.143331
C -1.228360 0.234935 -1.123705
C -2.701043 -0.100103 -0.815502
H -3.320774 0.777291 -1.024712
C -0.743395 1.371838 -0.226562
O -1.305106 2.461525 -0.272578
S -3.020682 -0.676715 0.908846
H -3.031327 -0.918335 -1.468002
H -3.075019 0.557538 1.469251
N 0.310709 1.117611 0.586584
H 0.581166 1.902764 1.168740
H 0.469193 -0.593120 1.798682
C 0.912106 -0.178948 0.874553
C 2.422177 -0.056059 1.123460
H 2.802345 -1.020402 1.471377
C 0.620488 -1.223140 -0.202706
O 1.255127 -2.272073 -0.236541
S 3.418944 0.504367 -0.322940
H 2.600640 0.685671 1.913303
H 3.422883 -0.692444 -0.961146
N -0.364148 -0.936145 -1.091345
H -0.534460 -1.670444 -1.769617

Conformer #4 in terms of energy, obtained from the XRD structure, as described in detail, energy -1291.12983129 a.u.
H 1.804719 -1.037358 -0.917169
H 1.879356 1.408220 -0.441631
H 1.871834 0.436839 -2.880830
S 0.491018 -0.973210 3.164929
H 0.105276 -2.130109 2.570748
N 1.071787 0.816084 -0.280187
C 0.935436 -0.381961 -1.096511
H 0.853096 -0.993540 -3.156154
C 0.931101 -0.053161 -2.600314
O 0.502849 2.264741 1.366669
C 0.281967 1.234392 0.740891
H -1.804719 1.037358 0.917169
H -1.879356 -1.408220 0.441631
H -1.871834 -0.436839 2.880830
S -0.491018 0.973210 -3.164929
H -0.105276 2.130109 -2.570748
N -1.071787 -0.816084 0.280187
C -0.935436 0.381961 1.096511
H -0.853096 0.993540 3.156154
C -0.931101 0.053161 2.600314
O -0.502849 -2.264741 -1.366669
C -0.281967 -1.234392 -0.740891

Conformer #5 in terms of energy, obtained from Conf9 structure, energy -1291.12577931 a.u.
H 2.304342 0.691642 1.294781
C 1.603973 0.452484 0.485511
C 2.428425 0.040813 -0.752459
H 1.776856 -0.176282 -1.607357
C 0.860624 1.756056 0.180059
O 1.465701 2.817939 0.110931
S 3.541457 -1.397573 -0.458332
H 3.086864 0.873943 -1.024284
H 2.587156 -2.360816 -0.505422
N -0.483982 1.648594 -0.014575
H -0.935781 2.508873 -0.304932
H -1.104145 0.037826 -1.246422
C -1.208499 0.388811 -0.205140
C -2.702464 0.592566 0.083483
H -3.034239 1.535999 -0.363639
C -0.621640 -0.705693 0.696018
O -1.314692 -1.615267 1.132490
S -3.757043 -0.763871 -0.600455
H -2.870454 0.641317 1.164301
H -4.394697 -0.004348 -1.527079
N 0.707573 -0.592840 0.963908
H 1.097742 -1.326923 1.543098

Conformer #6 in terms of energy, obtained from Conf2 structure, energy -1291.12514928 a.u.
H 2.199748 0.551380 1.928657
C 1.724189 0.154064 1.017556
C 2.813192 -0.631699 0.260650
H 3.702464 -0.002952 0.151446
C 1.306644 1.392808 0.226124
O 2.131525 2.264271 -0.025118
S 2.305323 -1.262579 -1.398638
H 3.088395 -1.519062 0.844300
H 2.555220 -0.114346 -2.076526
N 0.001710 1.476258 -0.147972
H -0.202939 2.283540 -0.727540
H -0.844643 -0.199047 -1.123596
C -0.953464 0.363697 -0.182562
C -2.386674 0.910615 -0.096440
H -2.475491 1.787130 -0.747486
C -0.679919 -0.630422 0.946810
O -1.566972 -1.352130 1.384091
S -3.657674 -0.322664 -0.633373
H -2.610179 1.217736 0.930927
H -4.151585 0.414868 -1.660174
N 0.594675 -0.669629 1.423680
H 0.741692 -1.353271 2.157598

Conformer #7 in terms of energy, obtained from Conf4 structure, energy -1291.12436938 a.u.
H -2.176870 0.677834 1.505023
C -1.501499 0.563228 0.649290
C -2.332704 0.572852 -0.657496
H -2.830834 1.547983 -0.733630
C -0.748955 -0.747650 0.883837
O -1.268728 -1.689492 1.463834
S -3.570521 -0.771858 -0.866742
H -1.666020 0.477159 -1.524628
H -4.174407 -0.633047 0.340146
N 0.526066 -0.778296 0.403309
H 0.986253 -1.681218 0.442232
H 1.086548 0.193957 -1.389277
C 1.218531 0.295045 -0.296966
C 2.726694 0.265142 -0.002709
H 2.913973 0.420580 1.065038
C 0.666218 1.679442 0.077853
O 1.316886 2.696661 -0.124923
S 3.565466 -1.287988 -0.546984
H 3.200061 1.083190 -0.552709
H 3.434779 -1.980185 0.612025
N -0.570127 1.693252 0.652448
H -0.939162 2.620902 0.830954

Conformer #8 in terms of energy, obtained from Conf3 structure, energy -1291.12432157 a.u.

H 1.807881 0.323018 -1.764630
C 1.315374 0.394870 -0.788465
C 2.334567 0.913299 0.252741
H 2.694797 1.894975 -0.080388
C 0.779299 -1.011899 -0.499417
O 1.293399 -2.008601 -0.984513
S 3.764851 -0.189127 0.609464
H 1.843217 1.055679 1.223682
H 4.103958 -0.447453 -0.678519
N -0.308058 -1.046675 0.319783
H -0.727710 -1.960240 0.444097
H -0.550315 0.466618 1.771607
C -1.017284 0.122579 0.828206
C -2.474370 -0.199666 1.177541
H -2.937978 0.695910 1.600290
C -0.917062 1.316665 -0.135668
O -1.774374 2.191371 -0.171129
S -3.511207 -0.824101 -0.211877
H -2.497866 -0.989578 1.939591
H -3.582868 0.357743 -0.873977
N 0.196227 1.331834 -0.918166
H 0.296047 2.141607 -1.518971

Conformer #9 in terms of energy, obtained from Conf1 structure, energy -1291.11991151 a.u.

H -2.242686 -0.922279 -1.398161
C -1.494576 -0.635902 -0.650313
C -2.066753 -0.913881 0.760600
H -2.321227 -1.979654 0.817287
C -1.190419 0.842539 -0.923024
O -2.016395 1.592048 -1.422005
S -3.512586 0.102986 1.275453
H -1.302207 -0.719122 1.523096
H -4.229855 -0.029325 0.131600
N 0.071017 1.231244 -0.580556
H 0.277365 2.215341 -0.703404
H 0.895034 0.264477 1.118467
C 1.075286 0.359045 0.034191
C 2.479956 0.941697 -0.166797
H 2.456919 2.024110 0.000957
C 0.973306 -1.052713 -0.571073
O 1.955899 -1.755106 -0.761203
S 3.737678 0.211349 0.972741
H 2.819723 0.754099 -1.190384
H 3.828109 1.280438 1.804000
N -0.295570 -1.443169 -0.882367
H -0.387611 -2.374211 -1.269942
